# Integrated Multi-Class Classification and Prediction of GPCR Allosteric Modulators by Machine Learning Intelligence

**DOI:** 10.3390/biom11060870

**Published:** 2021-06-11

**Authors:** Tianling Hou, Yuemin Bian, Terence McGuire, Xiang-Qun Xie

**Affiliations:** 1 Department of Pharmaceutical Sciences, Computational Chemical Genomics Screen (CCGS) Center and Pharmacometrics System Pharmacology Program, School of Pharmacy, University of Pittsburgh, Pittsburgh, PA 15261, USA; tih48@pitt.edu (T.H.); yuemin.bian@pitt.edu (Y.B.); tfm1@pitt.edu (T.M.); 2 NIH National Center of Excellence for Computational Drug Abuse Research (CDAR), University of Pittsburgh, Pittsburgh, PA 15261, USA; 3 Drug Discovery Institute, Departments of Computational Biology and of Structural Biology, University of Pittsburgh, Pittsburgh, PA 15261, USA

**Keywords:** GPCRs, allosteric regulation, machine learning, finger-prints, drug design

## Abstract

G-protein-coupled receptors (GPCRs) are the largest and most diverse group of cell surface receptors that respond to various extracellular signals. The allosteric modulation of GPCRs has emerged in recent years as a promising approach for developing target-selective therapies. Moreover, the discovery of new GPCR allosteric modulators can greatly benefit the further understanding of GPCR cell signaling mechanisms. It is critical but also challenging to make an accurate distinction of modulators for different GPCR groups in an efficient and effective manner. In this study, we focus on an 11-class classification task with 10 GPCR subtype classes and a random compounds class. We used a dataset containing 34,434 compounds with allosteric modulators collected from classical GPCR families A, B, and C, as well as random drug-like compounds. Six types of machine learning models, including support vector machine, naïve Bayes, decision tree, random forest, logistic regression, and multilayer perceptron, were trained using different combinations of features including molecular descriptors, Atom-pair fingerprints, MACCS fingerprints, and ECFP6 fingerprints. The performances of trained machine learning models with different feature combinations were closely investigated and discussed. To the best of our knowledge, this is the first work on the multi-class classification of GPCR allosteric modulators. We believe that the classification models developed in this study can be used as simple and accurate tools for the discovery and development of GPCR allosteric modulators.

## 1. Introduction

G-protein-coupled receptors (GPCRs) are the largest family of membrane proteins in the human genome and regulate a variety of extracellular signal transduction pathways, including photons, ions, hormones, neurotransmitters, odorants, and other stimuli [1,2]. Based on similarity and diversity of amino acid sequences and functions, GPCRs can be categorized into three main subfamilies, termed A, B, and C. As GPCRs are therapeutic targets for a broad spectrum of diseases, they have been of long-standing interest as therapeutic targets, and account for ~34% of the global market share of therapeutic drugs [3,4]. Most of the GPCR-targeted drugs are functionally active by binding to the orthosteric site of the receptor, which is the pocket bound by endogenous activating ligand [5]. However, an increasing number of drugs targeting the orthosteric sites have been withdrawn from the market due to low efficacy and undesired side effects [6,7]. The major issue that is associated with the orthosteric ligand is that their binding sites are often highly conserved across a single GPCR subfamily, making it difficult to achieve high selectivity for specific GPCR subtypes [8]. In recent years, great attention has been devoted to the discovery of drugs targeting GPCRs as allosteric modulators [9,10,11]. These small molecules bind to a site (allosteric site) that is topographically distinct from the orthosteric site of the GPCR protein and thus do not compete with orthosteric ligands [12]. Compared to the highly conserved orthosteric sites, allosteric binding pockets are more diverse across the same subfamily of GPCRs. This mechanism allows allosteric modulators to confer subtype selectivity. Meanwhile, the allosteric modulators also show a preferable safety profile due to the ‘ceiling’ effect [5,13,14]. In addition, Yiran Wu et al. added that the distinct pathways and allosteric pockets may enable allosteric modulators’ cooperativities among different protein subtypes [15]. Therefore, it is important to develop allosteric modulators as both therapeutic agents and research tools to bring new opportunities to drug discovery towards GPCRs and have an in-depth understanding of receptor modulation mechanisms.

Drug discovery is expensive. The process requires molecule design, lead optimization, in vitro and in vivo data analysis [16]. High-throughput screening (HTS) is a modern technique that is commonly used to facilitate the discovery of the allosteric modulators of GPCRs [17]. However, it is costly and often has been plagued with problems of high false-positive rates [18]. Alternatively, computational approaches, including homology modeling, molecular docking, and molecular dynamics simulation have been applied to aid drug discovery of novel allosteric modulators [19,20,21,22]. Yet, developing in silico screening methods that attain high accuracy remains challenging. At present, there is still an urgent demand for computational tools that can identify allosteric drugs from inactive random compounds and increase the chances of success in the development of allosteric modulators as lead clinical compounds.

While there are many traditional cheminformatic tools to assist the development of allosteric modulators, there have been few examples using machine learning (ML). ML has emerged as a promising pillar to promote data-driven decision-making, facilitate the process, and reduce the failure rates in drug discovery and development [23,24,25,26,27,28,29,30]. Kumar et al. developed multiple in silico models (Support Vector Machines (SVM), k-nearest neighbor algorithms, partial least square (PLS), etc.) to predict human intestinal absorption of diverse chemicals [31]. Jacob and Vert [32] used tensor-product-based features and applied SVM to predict protein-ligand interactions. Remarkably, in 2020, Google’s DeepMind’s AlphaFold made a scientific breakthrough for its astonishing performance on predicting protein 3D structures by using deep learning approaches [33,34].

We have previously reported an application of developing ML-based classification models for the prediction of orthosteric and allosteric regulations on cannabinoid receptors [13]. To expand the application to a broader scope of GPCR families and to handle a more diversified chemical space, in this paper, we proposed an 11-class classification task to discriminate allosteric modulators among different subtypes of GPCRs A, B, C subfamilies, and inactive compounds simultaneously. Diverse types of molecular features and multiple machine learning algorithms were applied for model training. The combinations of different types of molecular features and ML algorithms were carefully investigated to search which set of features works best for a specific classifier. The performance of trained ML models was systematically evaluated by using different metrics. This research gives the first report on the multi-class classification of GPCR allosteric modulators. The study can be of value for facilitating in silico screening and providing guidance for future discovery and development of GPCR allosteric modulators.

## 2. Materials and Methods

### 2.1. Data Collection and Preparation

In this study, the allosteric database (ASD) [35] was used for collecting GPCR allosteric modulators. Classical GPCR subfamilies A, B, and C were selected as targets to collect allosteric modulators. So far, the number of discovered allosteric modulators across different GPCR subtypes is of high variance. To construct a dataset that mimics this nature, we selected some common subtypes that are of a distinct number of allosteric modulators from each GPCR subtype. The number of collected allosteric modulators from each subtype is shown in Table 1. We also collected drug-like random compounds from the ZINC database [36] to serve as inactive decoy compounds. More than eight thousand drug-like compounds were randomly collected and integrated into the GPCR allosteric modulator datasets. Allosteric modulators collected from 10 GPCR subtypes were combined with the inactive compounds to make up the final dataset containing 34,434 compounds (Table 1).

### 2.2. Molecular Fingerprint and Descriptor Calculation

Both molecular descriptors and molecular fingerprints were used as molecular representations for all compounds in the datasets. A total of 119 molecular descriptors (ExactMW, SlogP, TPSA, NumHBD, NumHBA, etc.,), which characterize the physicochemical properties of the studied compounds were calculated using RDKit (http://www.rdkit.org/, accessed on 29 October 2020). Three different types of molecular fingerprints, Atom-pair fingerprints [37], MACCS fingerprints [38], and ECFP6 fingerprints [39] were calculated with a CDK toolkit [40]. Atom-pair fingerprints were encoded as standard bit vectors of length 1024 based on the atomic environments and shortest path separations of every atom pair in the molecule. MACCS fingerprints consist of 166-bit fingerprints representing the presence or absence of 166 substructural keys. ECFP6 are circular topological fingerprints that represent circular topological atom neighborhoods with 1024 descriptors.

### 2.3. Model Building

Here, six ML algorithms were employed to develop the classification models to discriminate between different subtypes of GPCR allosteric modulators and inactive drugs, including support vector machine (SVM), neural network/multilayer perceptron (MLP), decision tree (DT), random forest (RF), naïve Bayes (NB), and logistic regression. The open-source scikit-learn (http://scikitlearn.org/, accessed on 14 November 2020) was used for model building, tuning, validation, and result interpretation.

Support vector machine (SVM) [41] is a kernel-based algorithm widely used for binary classification and regression tasks. Each chemical structure was described as a binary string and worked as an eigenvector for SVM. The eigenvector was trained using the SVM algorithm, which results in a decision function for classification. The svm.SVC() method with three kernel functions (linear, rbf, poly) from scikit-learn was applied. The penalty parameter C and parameter γ for rbf and poly kernels were tuned on the training set by five-fold cross-validation using the grid search strategy.

Multilayer perceptron (MLP) [42] is a type of fully connected, feed-forward artificial neural network (ANN), consisting of three types of layers: the input layer, hidden layer, and output layer. An arbitrary number of hidden layers between the input and output layer is the true computational engine of the MLP. For each hidden layer, different numbers of hidden neurons can be assigned. MLP is based on calculating the values of hidden neurons in a current layer as the activated summation of weighted outputs of hidden neurons from a previous layer. The weights of the neuron connections are initially random but then adjusted through the backward propagation learning algorithm. Similar to SVM, we used grid search to optimize the hyperparameter for the MLPClassifier() method in scikit-learn. We searched for the optimal number of layers, number of hidden units, activation function (identity, logistic, tanh, ReLu), regularization parameter (0.0001, 0.001, 0.01, 0.1), and learning rate (0.1, 0.01, 0.001, 0.0001).

Decision tree (DT) [43] is a simple classic supervised learning method used to solve classification and regression problems. At each node of the tree, the attribute that gives the greatest information gain is chosen to make the decision. The data are divided in the most homogeneous way. Then the process is repeated on each smaller subset in a recursive manner. DecisionTreeClassifier() was applied for generating models with tuning on max_depth.

Random forest (RF) [44] is an ensemble method that leverages the power of a multitude of decision trees. In classification, regression, or other tasks, the final output is obtained by averaging the results of classification and regression trees that are grown on bootstrap samples. RandomForestClassifier() was applied. The best model was saved after the tuning on n_estimators (10, 50, 100) and max_depth (2, 3, 4, 5).

Naïve Bayes (NB) [45] is a simple probabilistic classifier based on Bayes’ theorem for conditional probability. This algorithm assumes that the attributes in a dataset are independent of each other. In other words, the NB classifier ignores the possible dependencies among the inputs and reduces a multivariate problem to a group of univariate problems.

Logistic regression (LR) [46] is a classification algorithm used for the prediction of the outcome of a categorical dependent variable from a set of predictor or independent variables. It is mainly used for prediction also calculating the probability of success. LogisticRegression() was applied and tuned with penalty (l1, l2, elasiticnet).

### 2.4. Chemical Space Analysis

The classification tasks in this study are completely implemented by machine intelligence without using any chemical or pharmacy domain knowledge. To investigate the possible relationships between the molecular properties or fingerprints and classification tasks, we visualized the chemical space distribution of each class by using the t-Distributed Stochastic Neighbor Embedding (t-SNE) method. Atom-pair, ECFP6, MACCS fingerprints, and molecular descriptors were used to represent molecules and were decomposed into two dimensions by t-SNE. The scikit-learn was applied for t-SNE analysis. Matplotlib library was used for plotting.

t-Distributed stochastic neighbor embedding (t-SNE) [47] is a nonlinear embedding technique developed by van der Maaten, L. and G. Hinton in 2008. It was used as a dimension reduction method well-suited for embedding high-dimensional data into a low-dimensional space of two or three dimensions [48]. t-SNE calculates the similarity measure between pairs of instances in the high dimensional space and the low dimensional space. The pairwise similarities of points were converted to joint probabilities. In this process, the Kullback–Leibler divergence between the joint probabilities of the low-dimensional embedding and the high-dimensional data was minimized. This neighbor embedding property makes t-SNE effective for identifying local clusters in the data.

### 2.5. Model Evaluation

In this study, the dataset was randomly divided into a training set (80%) and test set (20%) using a stratified sampling method. Then 80% of the training sets were randomly selected and used for training. The remaining compounds were used as a validation set. Various evaluation metrics were calculated to evaluate the performance of different ML models, including accuracy (ACC), balanced accuracy (Bal_ACC), precision, recall, f1-score, area under the receiver operating characteristic (ROC) curve (AUC) score, Cohen’s κ (CK) [49], and Matthews correlation coefficient (MCC) [50].

Micro-average and macro-average are two strategies being used for multiclass classification tasks. Here, we applied the macro-average method to calculate the precision, recall, and f1-score. Macro-average calculates each metric for each label separately and returns their unweighted mean. An ML model with good performance on macro-averaging metrics means it can recognize each class perfectly, even on small classes, which is a suitable case for our study. As for micro-averaging, we are aggregating the contributions of all classes to compute the average metric, which only emphasizes the performance of majority classes. The macro-averaged metric for each class is defined as:

$$\bar{M}=\overset{C}{\underset{i=1}{\sum }}M_{i}\cdot \frac{1}{C}$$

where *M* is the current metric, *C* is the number of total classes for the classification task, *i* denotes the *i*th class.

The following abbreviations are used for metric definitions: the number of true positives (TP), the number of false positives (FP), the number of true negatives (TN), and the number of false negatives (FN). The AUC score is one of the most widely used metrics that measures the overall performance of a classification model. It ranges between 0.5 and 1. A model with an AUC of 1 means perfect separation whereas an AUC of 0.5 means the model has no class separation capacity.

ACC is a measure of systematic error. It is the number of correctly predicted data points out of all data points.

$$\mathrm{ACC}\;=\frac{\mathrm{TP}+\mathrm{FN}}{\mathrm{TP}+\mathrm{TN}+\mathrm{FP}+\mathrm{FN}}$$

Recall is also called the true positive rate or sensitivity, which measures the ability of a classifier to find all of the positive samples.

$$\mathrm{recall}\;=\frac{\;\mathrm{TP}\;}{\;\mathrm{TP}\;+\;\mathrm{FN}\;}$$

Precision is known as the positive predictive value, which is the proportion of the predicted true label among all the retrieved instances.

$$\mathrm{precision}\;=\frac{\mathrm{TP}}{\mathrm{TP}+\mathrm{FP}}$$

The *f*_1_-score is a weighted average of the precision and recall and takes both false positives and false negatives into account.

$$f_{1}-\mathrm{score}=2\cdot \frac{\mathrm{precision}\cdot \mathrm{recall}}{\mathrm{precision}+\mathrm{recall}}$$

CK is used to estimate overall model performance by measuring the proximity of the predicted classes to the actual classes when compared to a random classification.

$$\mathrm{CK}\;=\frac{\mathrm{ACC}\;-\;\mathrm{Pe}}{1-\;\mathrm{Pe}}$$

where:

$$\mathrm{Pe}=\frac{\left(\mathrm{TP}+\mathrm{FP}\right)\times \left(\mathrm{TP}+\mathrm{FN}\right)+\left(\mathrm{TN}+\mathrm{FP}\right)\times \left(\mathrm{TN}+\mathrm{FN}\right)}{{\left(\mathrm{TP}+\mathrm{TN}+\mathrm{FP}+\mathrm{FN}\right)}^{2}}$$

When the dataset is imbalanced, Bal_ACC can be used to evaluate the general performance of an algorithm. It avoids inflated performance estimates and is computed as the average between sensitivity and specificity.

$$\mathrm{Bal}\text{\_}\mathrm{ACC}\;=\left[\frac{\mathrm{TP}}{\mathrm{TP}+\mathrm{FN}}+\frac{\mathrm{TN}}{\mathrm{TN}+\mathrm{FP}}\right]\cdot \frac{1}{n}$$

where *n* represents the total number of classes.

MCC is another useful metric when the dataset has varying classes and is imbalanced. It is a correlation coefficient between observed and predicted classes and has a range between −1 and 1. A value of −1 indicates a completely wrong prediction, while a coefficient of 1 indicates a perfect prediction.

$$\mathrm{MCC}=\frac{\mathrm{TP}\times \mathrm{TN}-\mathrm{FP}\times \mathrm{FN}}{\sqrt{\left(\mathrm{TP}+\mathrm{FP}\right)\left(\mathrm{TP}+\mathrm{FN}\right)\left(\mathrm{TN}+\mathrm{PP}\right)\left(\mathrm{TN}+\mathrm{FN}\right)}}$$

## 3. Results and Discussion

### 3.1. Overall Workflow

**Figure 1:**
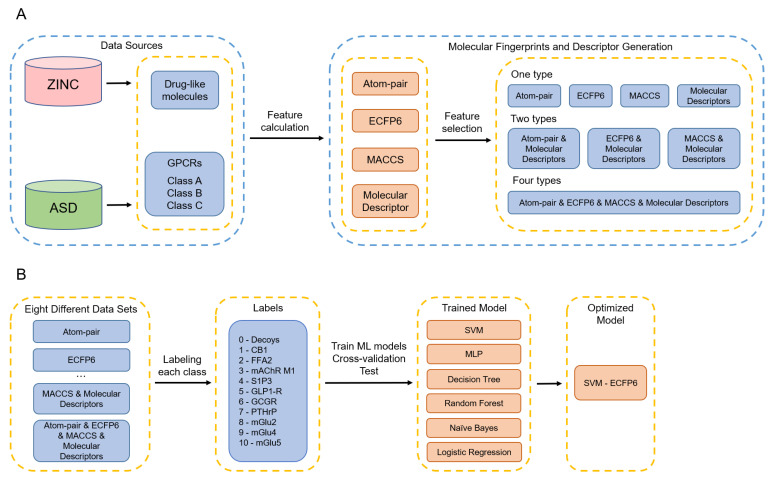
Schematic illustration of the workflow. (**A**) Workflow of data collection and reconstruction. (**B**) Workflow of model training and validation. GPCRs abbreviations: Cannabinoid receptor 1 (CB1); Free fatty acid receptor 2 (FFA2); Muscarinic acetylcholine receptor M1 (mAchR M1); Sphingosine 1-phosphate receptor 3 (S1P3); Glucagon-like peptide 1 receptor (GLP1-R); Glucagon receptor (GCGR); Parathyroid hormone-related peptide receptor (PTHrP); Metabotropic glutamate receptor 2 (mGlu2); Metabotropic glutamate receptor 4 (mGlu4); Metabotropic glutamate receptor 5 (mGlu5).

In this study, four subtypes from class A GPCRs, three subtypes from class B GPCRs, and three subtypes from class C GPCRs were collected from the ASD database. Inactive compounds were randomly selected from the ZINC database to cover a large drug-like chemical space. The allosteric modulators from 10 GPCRs subtypes and one random drug-like compounds class (decoys class) were used to construct the dataset for our 11-class classification task (Table 1, Figure 1A). Atom-pair, ECFP6, MACCS fingerprints, and molecular descriptors were calculated from the constructed dataset to represent four types of features. Different types of features can be used to evaluate the properties of the compounds from diverse aspects, which may affect the performance of ML models. Therefore, besides using one type of feature at each time for model training, we also paired fingerprints with molecular descriptors, as well as combining all four feature types to cover the best possible combinations (Figure 1A). Eight new datasets were generated from the different feature combinations. A total of 11 classes were labeled for classification from 0 to 10, respectively. Six supervised ML algorithms were applied to build classifiers for each dataset. We conducted a five-fold cross-validation for each dataset to select the best-performing model (Figure 1B). The ML models’ performance was evaluated by ACC, precision, recall, *f*1-score, MCC, CK, Bal_ACC, and AUC values.

### 3.2. Data Set Analysis

**Figure 2:**
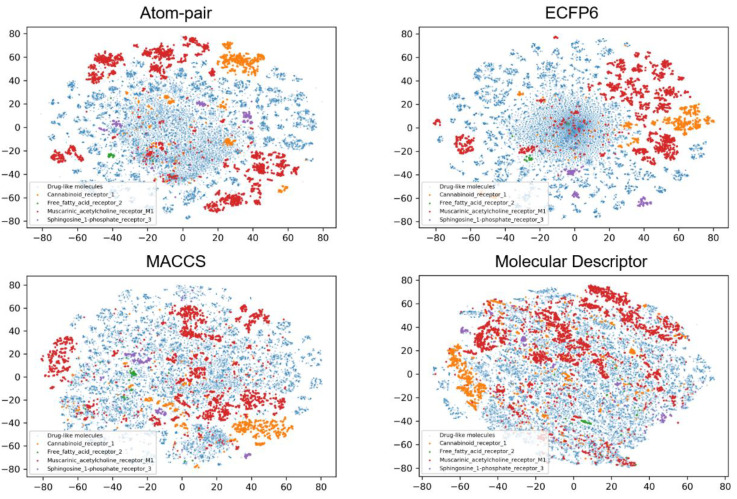
t-SNE method was used to visualize the chemical distribution of allosteric modulators from four subtypes of class A GPCRs according to Atom-pair, ECFP6, MACCS fingerprints, and molecular descriptors.

**Figure 3:**
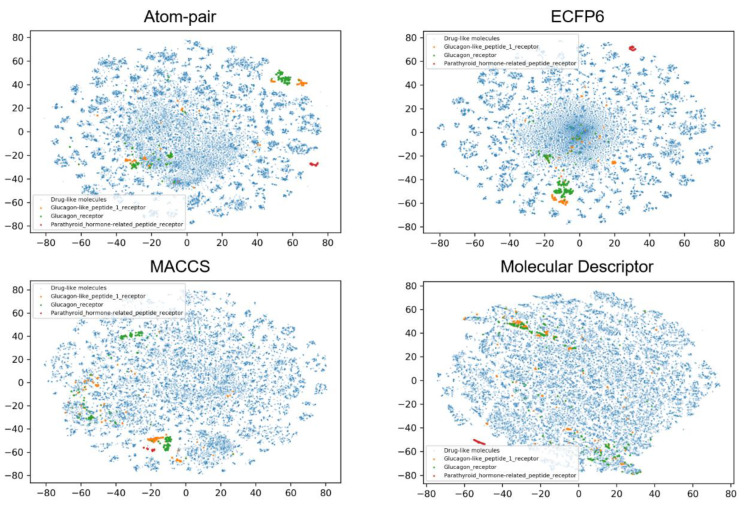
t-SNE method was used to visualize the chemical distribution of allosteric modulators from three subtypes of class B GPCRs according to Atom-pair, ECFP6, MACCS fingerprints, and molecular descriptors.

**Figure 4:**
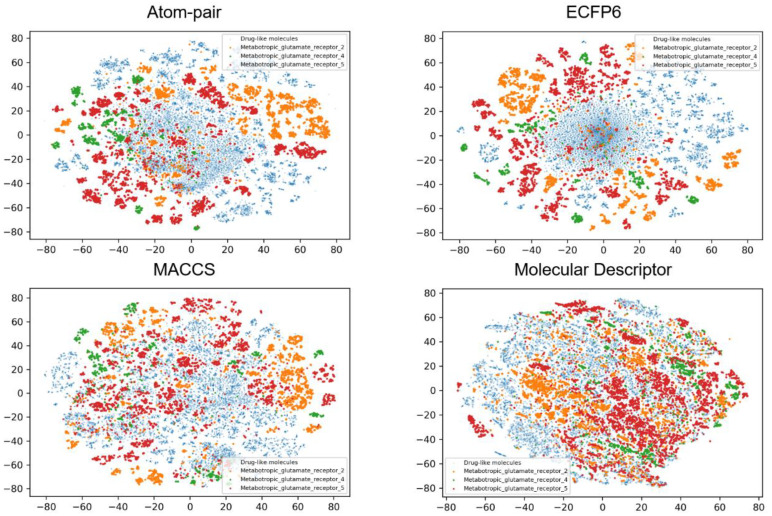
t-SNE method was used to visualize the chemical distribution of allosteric modulators from three subtypes of class C GPCRs according to Atom-pair, ECFP6, MACCS fingerprints, and molecular descriptors.

To investigate the possible relationships between the physicochemical properties or fingerprints and classification tasks, the chemical space distribution of each class was analyzed. t-SNE was used to decompose the molecular descriptors and fingerprints into two dimensions for visualizing. Figure 2, Figure 3 and Figure 4 show the results of the chemical space distribution of compounds in the dataset, which are represented by three types of fingerprints and molecular descriptors, respectively. As shown in Figure 2, Figure 3 and Figure 4, the blue dots represent drug-like molecules that define the background of the overall chemical property space. All 10 allosteric modulator subtypes fell in the defined chemical spaces (based on molecular fingerprints and properties), indicating that both similar and distinctive drug-like molecules were involved in the classification tasks for target-specific allosteric modulators. In comparison with known allosteric modulators, both similar and distinctive drug-like molecules were presented in the model training and validation processes. Similar molecules challenge the robustness of the model training process while distinctive molecules exhibit the vast chemical space to classifiers. Each of the allosteric modulator subtypes also occupies several specific regions which are distinct from one another, which may indicate their subtype selectivity. The distinct chemical space distribution of different allosteric subtypes shows the feasibility of applying our machine intelligence models.

### 3.3. Performance Evaluation on Different Feature Types

The ACC, precision, recall, *f*1-score, MCC, CK, Bal_ACC, and AUC values of all machine learning (ML) models on validation sets are summarized in Tables S1–S3. Radar charts are also plotted to visualize all the above metrics for both training sets and test sets (Figures S1–S4). The results on test sets are shown in Table 2, Table 3 and Table 4.

**Figure 5:**
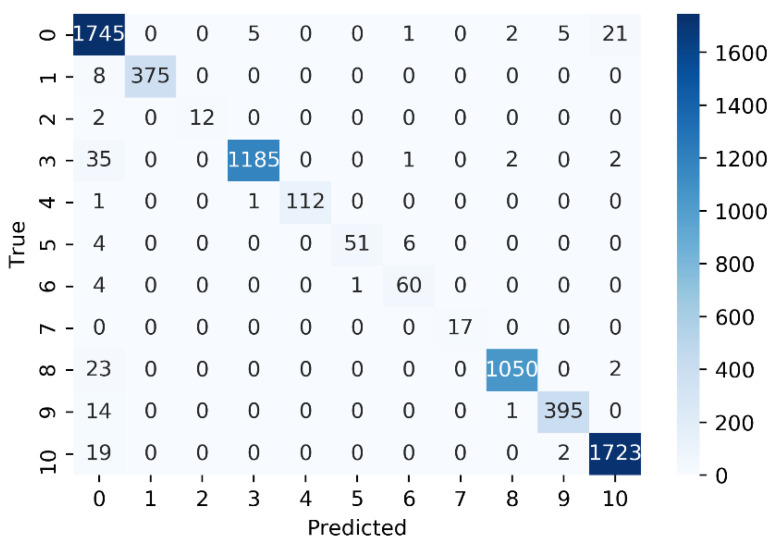
Confusion matrix (m) representing SVM classification performance on test set constructed with ECFP6 feature. The element m(i, j) is the number of times an observation of the ith true class was predicted to be of the jth class. Each colored cell of the confusion matrix chart corresponds to one element of the confusion matrix. Drug-like molecules, CB1, FFA2, mAchR M1, S1P3, GLP1-R, GCGR, PTHrP, mGlu2, mGlu4, and mGlu5 were labeled as 0 to 10, respectively.

According to the results, SVM trained on ECFP6 (SVM—ECFP6) outperformed other ML models with the highest scores on AUC, ACC, *f*1-score, CK, and MCC. It also showed satisfactory results on Bal_AUC (0.950), precision (0.978), and recall (0.950). The good result on Bal_AUC is exciting for an imbalanced dataset. The high *f*1-score of SVM—ECFP6 is also expected because it is a weighted average of precision and recall. MCC is a reliable and comprehensive assessment of the models’ performance. High MCC values mean that the ML model was able to correctly predict both the positive data instances as well as negative data instances, indicating an outstanding discriminant capability of the models’ performance. The confusion matrix is plotted to summarize the classification result of SVM—ECFP6 (Figure 5) on the test set. The results of all other models are shown in supplementary data (Figures S5–S16). As is shown in the confusion matrix, the separability of SVM—ECFP6 works well on all 11 classes, with most classes being correctly predicted. This model also shows a generalization capability even in small classes. Notably, a total of 17 test cases from the seventh class (PTHrP) was all correctly classified.

By comparing different ML algorithms, while SVM performed well when trained on one fingerprint type, its performance is significantly reduced when trained with molecular descriptors alone or in combination with other types of features. Compared to SVM, MLP shows a more stable performance on all datasets. When trained on datasets with two or more feature types, MLP showed superior overall performance, with AUC values above 0.95. It also showed the best performance on the molecular descriptor dataset, with an AUC value of 0.943. MLP can be considered a subset of deep neural networks (DNN), where its neural network architecture gives a competitive performance on high-dimensional datasets. This may result in the good performance of MLP on these datasets. RF has a balanced performance on most of the datasets. It generally shows good AUC scores but compared to SVM and MLP, the Bal_ACC and recall values are lower, indicating this classifier is more likely to be affected by the imbalanced dataset. LR also shows similar results as RF. NB and DT performed the worst among all ML models, which is also reasonable since they are more simple models and are often used as the baseline for comparison.

The selection of different features will also affect the ML models’ performance. As summarized in Table 5, models trained on the ECFP6 feature showed the best overall results; whereas, the molecular descriptors feature has poor performance compared with all other feature types or feature combinations. The results also showed that models trained on MACCS fingerprint underperformed the results of ECFP6 and Atom-pair fingerprint. The underperformance of MACCS could possibly be due to the fact that MACCS contains less information (166-bit) than ECFP6 (1024-bit) and Atom-pair (1024-bit), which would result in a less robust model. However, a fingerprint containing higher information density does not necessarily mean that it also should achieve a better outcome. Compared to ECFP6, which only contains structural information, Atom-pair is usually considered a hybrid type of fingerprint that contains both atomic and structural information. However, in our case, ECFP6 still shows the best outcome across all GPCR classes. Since there are 10 GPCR protein targets and one random decoy class involved in this study, the good result on ECFP6 means that ML models trained on this fingerprint can have a good impact on the majority of classes of selected GPCR protein targets. To obtain a deeper understanding of the predictive models, we further extracted the SlogP, molecular weight (M.W.), hydrogen bond acceptor (HBA), and hydrogen bond donor (HBD) from the molecular descriptors and conducted pair-wise distribution comparisons on each GPCR class with drug-like molecules, which are shown in Figures S17–S26 in the Supplementary Data. The comparison plots showed that most classes (all except the PTHrP) generally follow Lipinski’s rule of five since the distributions of drug-like molecules overlap with the target GPCR classes. However, the physicochemical properties of allosteric modulators from some of the GPCR classes do form distinguishable distributions from that of drug-like molecules. For example, the distribution of CB1 on M.W. is more concentrated than drug-like molecules with a mode around 500 whereas the mode of drug-like molecules is around 400. The distributions of GLP1-R and GCGR on SlogP are around 5, which is also different from the mode of SlogP for drug-like molecules that is around 4. It is worth noting that compared to the performance on datasets combining two feature types, MLP, LR, and RF all showed enhanced performance when trained on the dataset with all four feature types’ combinations, which is possibly due to the fact that the four feature types can give complementary information that is favored by these ML algorithms.

### 3.4. Performance Evaluation on Individual GPCR Classes

The *f*1-score takes the imbalanced data distribution into account and is the harmonic mean of precision and recall. Here we selected the *f*1-score as a representative metric to evaluate the ML models’ performance on one feature type (Table 6), two feature types (Table 7), and four feature types (Table 8) for each GPCR class. The *f*1-scores across different GPCR families were also compared and shown in supporting information (Table S4).

From Table 6, we can see that the best model SVM—ECFP6 has generally a high *f*1-score across all GPCR classes. It has an *f*1-score over 0.98 on CB1, mAchR M1, S1P3, PTHrP, mGlu2, and mGlu5. The *f*1-scores on FFA2, GLP1-R, GCGR of SVM—ECFP6 are all over 0.90 but below 0.95. The small sample sizes they have could impede the training process for robust models. PTHrP has a similar sample size (88 samples in total) to FFA2 (89 samples in total). Surprisingly, it has an *f*1-score of 1, indicating a precise and robust classification. The 88 samples from PTHrP are all polypeptides and share a core scaffold. They are very focused chemicals with small modifications to the core scaffolds. The highly distinguishable pattern may explain the good performance of the ML models in this class. While a large sample size is appreciated for building ML-based classification models, the current challenge remains on limited numbers of available GPCR allosteric modulators. In this study, the SVM-ECFP6 gives a satisfying performance across 11 classes (including small classes with around 90 samples), showing the feasibility of our method to be generalized to broader GPCR families.

In compliance with the previous observation, SVM and MLP have better performance on most classes than other ML models when trained with one feature type (except for molecular descriptors feature). When trained with two or more feature types, the SVM’s performance is significantly reduced while MLP, LR, and RF show better performance in each class (Table 6, Table 7 and Table 8). Moreover, similar to the result from SVM-ECFP6, many other models did not perform well on FFA2, GLP1-R, and GCGR, but most models have a high *f*1-score on PTHrP. In general, ML models trained on GPCR family A and C show a better *f*1-score than GPCR family B (Table S4) as all datasets for class B GPCRs have small numbers of instances.

## 4. Conclusions

In this study, four types of features, Atom-pair, ECFP6, MACCS fingerprints, and molecular descriptors were used to construct a series of datasets. The chemical space analysis of all four features demonstrated that the 10 allosteric subtypes form spatial patterns that are distinguishable from each other. Six ML models were built and trained on datasets with different feature combinations. SVM—ECFP6 shows the best results (AUC = 0.974, ACC = 0.976, Bal_ACC = 0.950, *f*1-score = 0.963, CK = 0.971, MCC = 0.971, precision = 0.978, recall = 0.950). MLP has the most stable performance across different feature combinations. In particular, it outperformed other ML models on datasets constructed with two or more features. By comparing the ML model’s performance on different features, we found that when only using one feature type for training, ECFP6 is the best choice for its good performance on most ML models. Mixed effects were seen on datasets with various feature combinations on different ML models. In the field of drug discovery, we need to frequently deal with imbalanced datasets [51]. The model developed in our study shows a good generalization capability on an imbalanced dataset. To the best of our knowledge, this study is the first work on the multi-class classification of GPCR allosteric modulators. The developed multi-class classifiers provide alternative options on virtual screening besides the conventional structure-based and ligand-based methods. Besides being of benefit to potential hit identification campaigns on GPCR allosteric modulators, this study can also be of value to demonstrate the possibility of adapting machine learning to the broad area of drug discovery.

## Figures and Tables

**Table 1:** Dataset Information.

Dataset	Training Set	Validation Set	Test Set	Total
Drug-like compounds	5691	1423	1779	8893
CB1—Cannabinoid receptor 1 (Class A)	1242	311	383	1936
FFA2—Free fatty acid receptor 2 (Class A)	60	15	14	89
mAchR M1—Muscarinic acetylcholine receptor M1 (Class A)	4019	1005	1225	6249
S1PR3—Sphingosine 1-phosphate receptor 3 (Class A)	323	81	114	518
GLP1-R—Glucagon-like peptide 1 receptor (Class B)	202	51	61	314
GCGR—Glucagon receptor (Class B)	285	72	65	422
PTHrP—Parathyroid hormone/parathyroid hormone-related peptide receptor (Class B)	56	15	17	88
mGlu2—Metabotropic glutamate receptor 2 (Class C)	3519	880	1075	5474
mGlu4—Metabotropic glutamate receptor 4 (Class C)	1342	336	410	2088
mGlu5—Metabotropic glutamate receptor 5 (Class C)	5295	1324	1744	8363

**Table 2:** Results of the Test Set on Each Dataset with One Feature Type.

Datasets	Model	AUC	ACC	Bal_ACC	*f*_1_-Score	CK	MCC	Precision	Recall
Atom-pair	SVM	0.966	0.972	0.936	0.949	0.965	0.965	0.965	0.936
	NB	0.820	0.627	0.679	0.625	0.550	0.559	0.644	0.679
	MLP	0.960	0.954	0.925	0.933	0.943	0.943	0.944	0.925
	LR	0.942	0.912	0.895	0.907	0.891	0.891	0.922	0.895
	RF	0.942	0.956	0.890	0.925	0.946	0.946	0.971	0.890
	DT	0.861	0.770	0.748	0.743	0.715	0.716	0.743	0.748
ECFP6	SVM	0.974	0.976	0.950	0.963	0.971	0.971	0.978	0.950
	NB	0.916	0.868	0.847	0.872	0.835	0.839	0.911	0.847
	MLP	0.957	0.958	0.919	0.931	0.948	0.948	0.948	0.919
	LR	0.955	0.940	0.916	0.932	0.925	0.925	0.954	0.916
	RF	0.946	0.964	0.897	0.931	0.956	0.956	0.980	0.897
	DT	0.896	0.822	0.812	0.789	0.780	0.780	0.771	0.812
MACCS	SVM	0.961	0.963	0.926	0.934	0.954	0.954	0.944	0.926
	NB	0.822	0.629	0.684	0.588	0.551	0.555	0.562	0.684
	MLP	0.954	0.940	0.914	0.915	0.925	0.925	0.918	0.914
	LR	0.898	0.839	0.815	0.834	0.800	0.800	0.857	0.815
	RF	0.945	0.961	0.895	0.924	0.951	0.951	0.962	0.895
	DT	0.890	0.854	0.796	0.791	0.820	0.820	0.790	0.796
Molecular Descriptors	SVM	0.809	0.788	0.644	0.682	0.734	0.736	0.751	0.644
	NB	0.824	0.618	0.690	0.576	0.538	0.541	0.531	0.690
	MLP	0.943	0.936	0.893	0.893	0.920	0.920	0.902	0.893
	LR	0.887	0.840	0.793	0.810	0.801	0.801	0.834	0.793
	RF	0.941	0.953	0.887	0.919	0.941	0.941	0.966	0.887
	DT	0.880	0.828	0.779	0.767	0.787	0.788	0.758	0.779

**Table 3:** Results of the Test Set on Each Dataset with Two Feature Types.

Datasets	Model	AUC	ACC	Bal_ACC	*f*_1_-Score	CK	MCC	Precision	Recall
Atom-pair and Molecular Descriptors	SVM	0.825	0.818	0.672	0.715	0.771	0.773	0.791	0.672
	NB	0.834	0.660	0.704	0.667	0.588	0.597	0.688	0.704
	MLP	0.959	0.945	0.925	0.918	0.932	0.932	0.914	0.925
	LR	0.959	0.947	0.923	0.931	0.934	0.934	0.941	0.923
	RF	0.944	0.962	0.894	0.927	0.952	0.953	0.975	0.894
	DT	0.875	0.827	0.770	0.765	0.785	0.785	0.762	0.770
ECFP6 and Molecular Descriptors	SVM	0.819	0.809	0.661	0.703	0.759	0.761	0.776	0.661
	NB	0.922	0.871	0.859	0.875	0.838	0.841	0.901	0.859
	MLP	0.973	0.960	0.951	0.945	0.950	0.950	0.939	0.951
	LR	0.960	0.961	0.925	0.939	0.952	0.952	0.957	0.925
	RF	0.949	0.968	0.902	0.932	0.960	0.960	0.978	0.902
	DT	0.876	0.814	0.773	0.759	0.770	0.770	0.747	0.773
MACCS and Molecular Descriptors	SVM	0.812	0.793	0.649	0.688	0.739	0.741	0.760	0.649
	NB	0.853	0.659	0.743	0.621	0.588	0.592	0.579	0.743
	MLP	0.943	0.951	0.891	0.913	0.939	0.939	0.950	0.891
	LR	0.922	0.897	0.856	0.871	0.872	0.872	0.896	0.856
	RF	0.945	0.961	0.895	0.926	0.951	0.951	0.971	0.895
	DT	0.894	0.837	0.806	0.785	0.799	0.799	0.767	0.806

**Table 4:** Results of the Test Set on Dataset with Four Feature Types.

Datasets	Model	AUC	ACC	Bal_ACC	*f*_1_-Score	CK	MCC	Precision	Recall
Atom-pair and ECFP6 and MACCS and Molecular Descriptors	SVM	0.837	0.841	0.693	0.735	0.800	0.802	0.807	0.693
	NB	0.884	0.776	0.792	0.778	0.725	0.729	0.800	0.792
	MLP	0.962	0.967	0.941	0.941	0.960	0.960	0.958	0.928
	LR	0.968	0.967	0.940	0.950	0.959	0.959	0.962	0.940
	RF	0.950	0.970	0.904	0.933	0.962	0.962	0.976	0.904
	DT	0.883	0.823	0.787	0.774	0.781	0.781	0.765	0.787

**Table 5:** Overall Performance Comparison of Different Feature Types (Average over Nine Datasets on All ML Models).

Datasets	AUC	ACC	Bal_ACC	*f*_1_-Score	CK	MCC	Precision	Recall
Atom-pair	0.915	0.865	0.846	0.847	0.835	0.837	0.865	0.846
ECFP6	0.941	0.921	0.890	0.903	0.903	0.903	0.924	0.890
MACCS	0.912	0.864	0.838	0.831	0.834	0.834	0.839	0.838
Molecular Descriptors	0.881	0.827	0.781	0.775	0.787	0.788	0.790	0.781
Atom-pair and Molecular Descriptors	0.899	0.860	0.815	0.821	0.827	0.829	0.845	0.815
ECFP6 and Molecular Descriptors	0.917	0.897	0.845	0.859	0.872	0.872	0.883	0.845
MACCS and Molecular Descriptors	0.895	0.850	0.807	0.801	0.815	0.816	0.821	0.807
Atom-pair and ECFP6 and MACCS and Molecular Descriptors	0.914	0.891	0.843	0.852	0.865	0.866	0.878	0.841

**Table 6:** The *f*_1_-score of the Test Set on Each Dataset with One Feature Type on Each GPCR Class.

Datasets	Model	Drug-Like	CB1	FFA2	mAchR M1	S1P3	GLP1-R	GCGR	PTHrP	mGlu2	mGlu4	mGlu5
Atom-pair	SVM	0.954	0.987	0.889	0.983	0.991	0.822	0.870	1.000	0.979	0.979	0.981
	NB	0.609	0.683	0.373	0.715	0.720	0.456	0.673	0.919	0.676	0.387	0.661
	MLP	0.928	0.967	0.923	0.971	0.974	0.789	0.838	1.000	0.956	0.940	0.975
	LR	0.869	0.952	0.880	0.934	0.978	0.765	0.840	1.000	0.911	0.913	0.936
	RF	0.925	0.970	0.783	0.972	0.950	0.792	0.869	1.000	0.966	0.966	0.978
	DT	0.698	0.802	0.690	0.824	0.778	0.558	0.528	1.000	0.743	0.711	0.840
ECFP6	SVM	0.960	0.989	0.923	0.981	0.991	0.903	0.902	1.000	0.986	0.973	0.987
	NB	0.813	0.812	0.800	0.901	0.944	0.785	0.871	1.000	0.892	0.868	0.906
	MLP	0.934	0.979	0.750	0.968	0.987	0.885	0.847	1.000	0.973	0.944	0.971
	LR	0.902	0.980	0.833	0.955	0.978	0.875	0.885	1.000	0.954	0.942	0.952
	RF	0.938	0.973	0.783	0.976	0.982	0.784	0.878	1.000	0.979	0.967	0.982
	DT	0.757	0.847	0.625	0.848	0.861	0.618	0.645	1.000	0.836	0.754	0.885
MACCS	SVM	0.943	0.978	0.815	0.975	0.973	0.847	0.826	1.000	0.978	0.967	0.972
	NB	0.612	0.648	0.275	0.665	0.719	0.317	0.438	1.000	0.695	0.428	0.670
	MLP	0.909	0.965	0.929	0.956	0.964	0.714	0.789	1.000	0.952	0.930	0.960
	LR	0.799	0.882	0.720	0.849	0.942	0.729	0.775	1.000	0.834	0.754	0.885
	RF	0.936	0.976	0.696	0.976	0.964	0.841	0.866	1.000	0.972	0.959	0.974
	DT	0.791	0.867	0.500	0.876	0.793	0.643	0.653	0.971	0.875	0.824	0.913
Molecular Descriptors	SVM	0.759	0.812	0.000	0.836	0.865	0.465	0.566	1.000	0.787	0.568	0.839
	NB	0.677	0.570	0.329	0.671	0.752	0.470	0.406	0.829	0.576	0.402	0.652
	MLP	0.909	0.938	0.733	0.960	0.965	0.752	0.759	1.000	0.949	0.908	0.955
	LR	0.828	0.880	0.583	0.881	0.900	0.766	0.705	1.000	0.838	0.669	0.860
	RF	0.925	0.969	0.727	0.971	0.959	0.811	0.870	1.000	0.966	0.943	0.969
	DT	0.784	0.846	0.483	0.856	0.787	0.626	0.653	0.941	0.823	0.753	0.886

**Table 7:** The *f*_1_-score of the Test Set on Each Dataset with Two Feature Types on Each GPCR Class.

Datasets	Model	Drug-Like	CB1	FFA2	mAchR M1	S1P3	GLP1-R	GCGR	PTHrP	mGlu2	mGlu4	mGlu5
Atom-pair and Molecular Descriptors	SVM	0.782	0.832	0.000	0.864	0.870	0.578	0.608	1.000	0.832	0.638	0.860
	NB	0.648	0.699	0.550	0.742	0.755	0.510	0.706	0.919	0.725	0.393	0.693
	MLP	0.922	0.944	0.889	0.965	0.911	0.828	0.837	0.971	0.953	0.912	0.967
	LR	0.926	0.964	0.846	0.965	0.974	0.870	0.859	1.000	0.952	0.928	0.957
	RF	0.936	0.972	0.727	0.975	0.968	0.811	0.898	1.000	0.975	0.952	0.979
	DT	0.763	0.843	0.500	0.861	0.773	0.627	0.580	0.941	0.825	0.817	0.887
ECFP6 and Molecular Descriptors	SVM	0.773	0.828	0.000	0.858	0.865	0.523	0.596	1.000	0.818	0.613	0.856
	NB	0.827	0.829	0.800	0.904	0.964	0.796	0.894	1.000	0.885	0.822	0.905
	MLP	0.941	0.979	0.963	0.975	0.961	0.828	0.866	1.000	0.960	0.942	0.975
	LR	0.942	0.981	0.833	0.974	0.965	0.862	0.901	0.971	0.968	0.956	0.971
	RF	0.944	0.977	0.727	0.978	0.987	0.808	0.899	1.000	0.979	0.973	0.983
	DT	0.753	0.882	0.364	0.832	0.850	0.592	0.609	1.000	0.818	0.776	0.869
MACCS and Molecular Descriptors	SVM	0.761	0.813	0.000	0.839	0.865	0.488	0.585	1.000	0.795	0.581	0.842
	NB	0.673	0.645	0.371	0.718	0.786	0.377	0.480	0.971	0.694	0.428	0.690
	MLP	0.930	0.958	0.727	0.968	0.954	0.800	0.837	1.000	0.967	0.933	0.964
	LR	0.878	0.923	0.696	0.929	0.922	0.815	0.806	1.000	0.894	0.804	0.916
	RF	0.937	0.971	0.727	0.973	0.968	0.833	0.875	1.000	0.973	0.954	0.977
	DT	0.780	0.848	0.500	0.873	0.827	0.591	0.696	1.000	0.846	0.786	0.888

**Table 8:** The *f*_1_-score of the Test Set on Dataset with Four Feature Types on Each GPCR Class.

Datasets	Model	Drug-Like	CB1	FFA2	mAchR M1	S1P3	GLP1-R	GCGR	PTHrP	mGlu2	mGlu4	mGlu5
Atom-pair and ECFP6 and MACCS and Molecular Descriptors	SVM	0.804	0.852	0.000	0.889	0.900	0.578	0.621	1.000	0.860	0.702	0.877
	NB	0.739	0.803	0.632	0.848	0.891	0.678	0.821	0.971	0.837	0.512	0.823
	MLP	0.953	0.981	0.800	0.982	0.991	0.865	0.870	1.000	0.970	0.964	0.975
	LR	0.953	0.979	0.880	0.981	0.974	0.881	0.894	1.000	0.972	0.961	0.973
	RF	0.948	0.979	0.727	0.979	0.982	0.833	0.882	1.000	0.981	0.973	0.985
	DT	0.753	0.869	0.500	0.849	0.830	0.596	0.617	1.000	0.841	0.775	0.882

## Data Availability

The data presented in this study are available in Supplementary Materials.

## Supplementary Materials

The following are available online at https://www.mdpi.com/article/10.3390/biom11060870/s1. Collected raw chemical datasets. Results of the validation set on each dataset with different feature types (Tables S1–S3); The f1-score among different GPCR families on each dataset (Table S4); Radar chart of validation sets on different feature types (Figures S1–S4); Confusion matrix of each ML model on different feature types (Figures S5–S16). Pair-wise distribution comparisons of each GPCR class with drug-like molecules on SlogP, molecular weight (M.W.), hydrogen bond acceptor (HBA), and hydrogen bond doner (HBD) (Figures S17–S26).

## References

[1] Ritter S.L., Hall R.A. (2009). Fine-tuning of GPCR activity by receptor-interacting proteins. Nat. Rev. Mol. Cell Biol..

[2] Raschka S., Kaufman B. (2020). Machine learning and AI-based approaches for bioactive ligand discovery and GPCR-ligand recognition. Methods.

[3] Congreve M., de Graaf C., Swain N.A., Tate C.G. (2020). Impact of GPCR Structures on Drug Discovery. Cell.

[4] Hauser A.S., Attwood M.M., Rask-Andersen M., Schioth H.B., Gloriam D.E. (2017). Trends in GPCR drug discovery: New agents, targets and indications. Nat. Rev. Drug Discov..

[5] Bridges T.M., Lindsley C.W. (2008). G-protein-coupled receptors: From classical modes of modulation to allosteric mechanisms. ACS Chem. Biol..

[6] Feng Z., Hu G., Ma S., Xie X.-Q. (2015). Computational Advances for the Development of Allosteric Modulators and Bitopic Ligands in G Protein-Coupled Receptors. AAPS J..

[7] Sloop K.W., Emmerson P.J., Statnick M.A., Willard F.S. (2018). The current state of GPCR-based drug discovery to treat metabolic disease. Br. J. Pharmacol..

[8] Conn P.J., Lindsley C.W., Meiler J., Niswender C.M. (2014). Opportunities and challenges in the discovery of allosteric modulators of GPCRs for treating CNS disorders. Nat. Rev. Drug Discov..

[9] Leach K., Sexton P.M., Christopoulos A. (2007). Allosteric GPCR modulators: Taking advantage of permissive receptor pharmacology. Trends Pharmacol. Sci..

[10] Lindsley C.W., Emmitte K.A., Hopkins C.R., Bridges T.M., Gregory K.J., Niswender C.M., Conn P.J. (2016). Practical Strategies and Concepts in GPCR Allosteric Modulator Discovery: Recent Advances with Metabotropic Glutamate Receptors. Chem. Rev..

[11] Nickols H.H., Conn P.J. (2014). Development of allosteric modulators of GPCRs for treatment of CNS disorders. Neurobiol. Dis..

[12] Bian Y., Jun J.J., Cuyler J., Xie X.-Q. (2020). Covalent allosteric modulation: An emerging strategy for GPCRs drug discovery. Eur. J. Med. Chem..

[13] Bian Y., Jing Y., Wang L., Ma S., Jun J.J., Xie X.-Q. (2019). Prediction of Orthosteric and Allosteric Regulations on Cannabinoid Receptors Using Supervised Machine Learning Classifiers. Mol. Pharm..

[14] Laprairie R.B., Bagher A.M., Kelly M.E., Denovan-Wright E.M. (2015). Cannabidiol is a negative allosteric modulator of the cannabinoid CB1 receptor. Br. J. Pharmacol..

[15] Wu Y., Tong J., Ding K., Zhou Q., Zhao S. (2019). GPCR Allosteric Modulator Discovery. Adv. Exp. Med. Biol..

[16] Schneider P., Schneider G. (2016). De Novo Design at the Edge of Chaos. J. Med. Chem..

[17] Zhang R., Xie X. (2012). Tools for GPCR drug discovery. Acta Pharmacol. Sin..

[18] Finak G., Gottardo R. (2016). Promises and Pitfalls of High-Throughput Biological Assays. Methods Mol. Biol..

[19] Evers A., Klabunde T. (2005). Structure-based drug discovery using GPCR homology modeling: Successful virtual screening for antagonists of the alpha1A adrenergic receptor. J. Med. Chem..

[20] Liu L., Jockers R. (2020). Structure-Based Virtual Screening Accelerates GPCR Drug Discovery. Trends Pharmacol. Sci..

[21] Petrucci V., Chicca A., Glasmacher S., Paloczi J., Cao Z., Pacher P., Gertsch J. (2017). Pepcan-12 (RVD-hemopressin) is a CB2 receptor positive allosteric modulator constitutively secreted by adrenals and in liver upon tissue damage. Sci. Rep..

[22] Wang H., Duffy R.A., Boykow G.C., Chackalamannil S., Madison V.S. (2008). Identification of novel cannabinoid CB1 receptor antagonists by using virtual screening with a pharmacophore model. J. Med. Chem..

[23] Bemister-Buffington J., Wolf A.J., Raschka S., Kuhn L.A. (2020). Machine Learning to Identify Flexibility Signatures of Class A GPCR Inhibition. Biomolecules.

[24] Bian Y., Wang J., Jun J.J., Xie X.-Q. (2019). Deep Convolutional Generative Adversarial Network (dcGAN) Models for Screening and Design of Small Molecules Targeting Cannabinoid Receptors. Mol. Pharm..

[25] Liang G., Fan W., Luo H., Zhu X. (2020). The emerging roles of artificial intelligence in cancer drug development and precision therapy. Biomed. Pharmacother.

[26] Ma C., Wang L., Xie X.-Q. (2011). Ligand Classifier of Adaptively Boosting Ensemble Decision Stumps (LiCABEDS) and its application on modeling ligand functionality for 5HT-subtype GPCR families. J. Chem. Inf. Model..

[27] Ma C., Wang L., Yang P., Myint K.Z., Xie X.-Q. (2013). LiCABEDS II. Modeling of ligand selectivity for G-protein-coupled cannabinoid receptors. J. Chem. Inf. Model..

[28] Reda C., Kaufmann E., Delahaye-Duriez A. (2020). Machine learning applications in drug development. Comput. Struct. Biotechnol. J..

[29] Tsou L.K., Yeh S.H., Ueng S.H., Chang C.P., Song J.S., Wu M.H., Chang H.F., Chen S.R., Shih C., Chen C.T. (2020). Comparative study between deep learning and QSAR classifications for TNBC inhibitors and novel GPCR agonist discovery. Sci. Rep..

[30] Bian Y., Xie X.-Q. (2021). Generative chemistry: Drug discovery with deep learning generative models. J. Mol. Model..

[31] Kumar R., Sharma A., Siddiqui M.H., Tiwari R.K. (2017). Prediction of Human Intestinal Absorption of Compounds Using Artificial Intelligence Techniques. Curr. Drug Discov. Technol..

[32] Jacob L., Vert J.P. (2008). Protein-ligand interaction prediction: An improved chemogenomics approach. Bioinformatics.

[33] AlQuraishi M. (2019). AlphaFold at CASP13. Bioinformatics.

[34] Senior A.W., Evans R., Jumper J., Kirkpatrick J., Sifre L., Green T., Qin C., Zidek A., Nelson A.W.R., Bridgland A. (2020). Improved protein structure prediction using potentials from deep learning. Nature.

[35] Shen Q., Wang G., Li S., Liu X., Lu S., Chen Z., Song K., Yan J., Geng L., Huang Z. (2016). ASD v3.0: Unraveling allosteric regulation with structural mechanisms and biological networks. Nucleic Acids Res..

[36] Irwin J.J., Shoichet B.K. (2005). ZINC--a free database of commercially available compounds for virtual screening. J. Chem. Inf. Model..

[37] Raymond E., Carhart D.H.S., Venkataraghavan R. (1985). Atom pairs as molecular features in structure-activity studies: Definition and applications. J. Chem. Inf. Comput. Sci..

[38] Durant J.L., Leland B.A., Henry D.R., Nourse J.G. (2002). Reoptimization of MDL keys for use in drug discovery. J. Chem. Inf. Comput. Sci..

[39] Rogers D., Hahn M. (2010). Extended-connectivity fingerprints. J. Chem. Inf. Model..

[40] Steinbeck C., Hoppe C., Kuhn S., Floris M., Guha R., Willighagen E.L. (2006). Recent developments of the chemistry development kit (CDK)—An open-source java library for chemo- and bioinformatics. Curr. Pharm. Des..

[41] Cortes C., Vapnik V. (1995). Support-Vector Networks. Mach. Learn..

[42] Isa N.A.M., Mamat W.M.F.W. (2011). Clustered-hybrid multilayer perceptron network for pattern recognition application. Appl. Soft Comput..

[43] Safavian S.R., Landgrebe D. (1991). A survey of decision tree classifier methodology. IEEE Trans. Syst. Man Cybern..

[44] Plewczynski D., von Grotthuss M., Rychlewski L., Ginalski K. (2009). Virtual high throughput screening using combined random forest and flexible docking. Comb. Chem. High Throughput Screen..

[45] Friedman N., Geiger D., Goldszmidt M. (1997). Bayesian Network Classifiers. Mach. Learn..

[46] Tolles J., Meurer W.J. (2016). Logistic Regression: Relating Patient Characteristics to Outcomes. JAMA.

[47] Van der Maaten L., Hinton G. (2008). Visualizing Data using t-SNE. J. Mach. Learn. Res..

[48] Zhou B., Jin W. (2020). Visualization of Single Cell RNA-Seq Data Using t-SNE in R. Methods Mol. Biol.

[49] Carletta J. (1996). Assessing Agreement on Classification Tasks: The Kappa Statistic. Comput. Linguist..

[50] Matthews B.W. (1975). Comparison of the predicted and observed secondary structure of T4 phage lysozymeBiochim. Biophys. Acta Protein Struct..

[51] Korkmaz S. (2020). Deep Learning-Based Imbalanced Data Classification for Drug Discovery. J. Chem. Inf. Model..

