# Supplementary material for: Integrated Multi-Class Classification and Prediction of GPCR Allosteric Modulators by Machine Learning Intelligence
Source: Biomolecules. 2021 Jun 11;11(6):870. doi: 10.3390/biom11060870 (PMC8230833; doi:10.3390/biom11060870)
Supplement: Supplementary file 1 [file biomolecules-11-00870-s001.zip › biomolecules-1213864-supplementary-conversion.pdf]

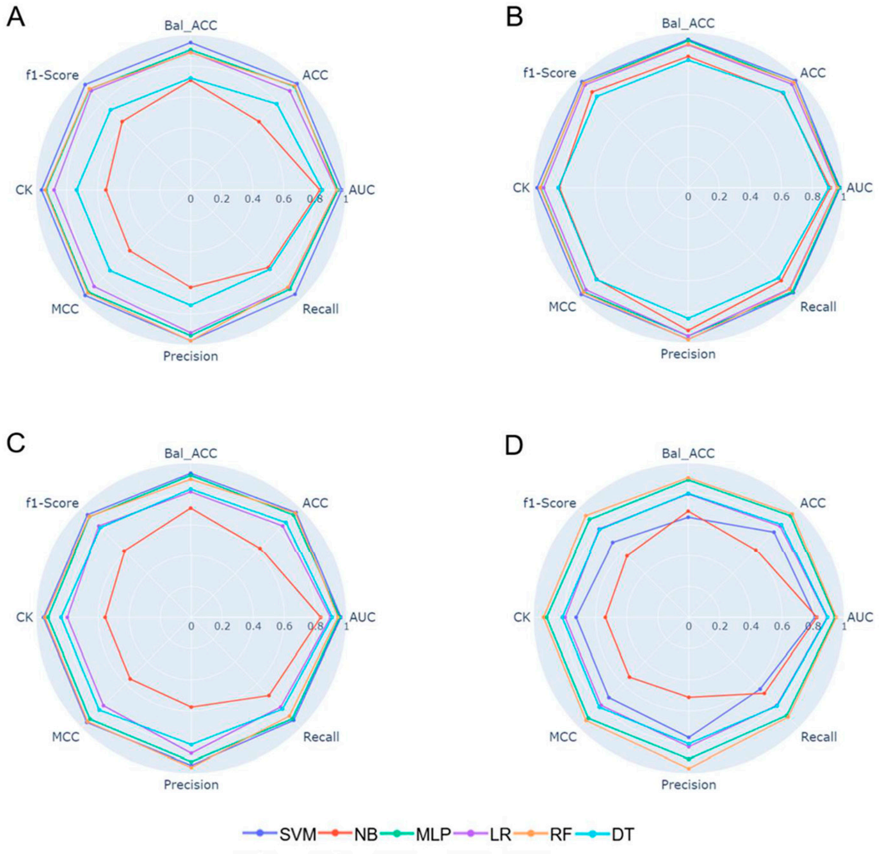

Figure S1. Radar chart of validation sets on Atom-pair (**A**), ECFP6 (**B**), MACCS fingerprints (C), and molecular descriptors (D).

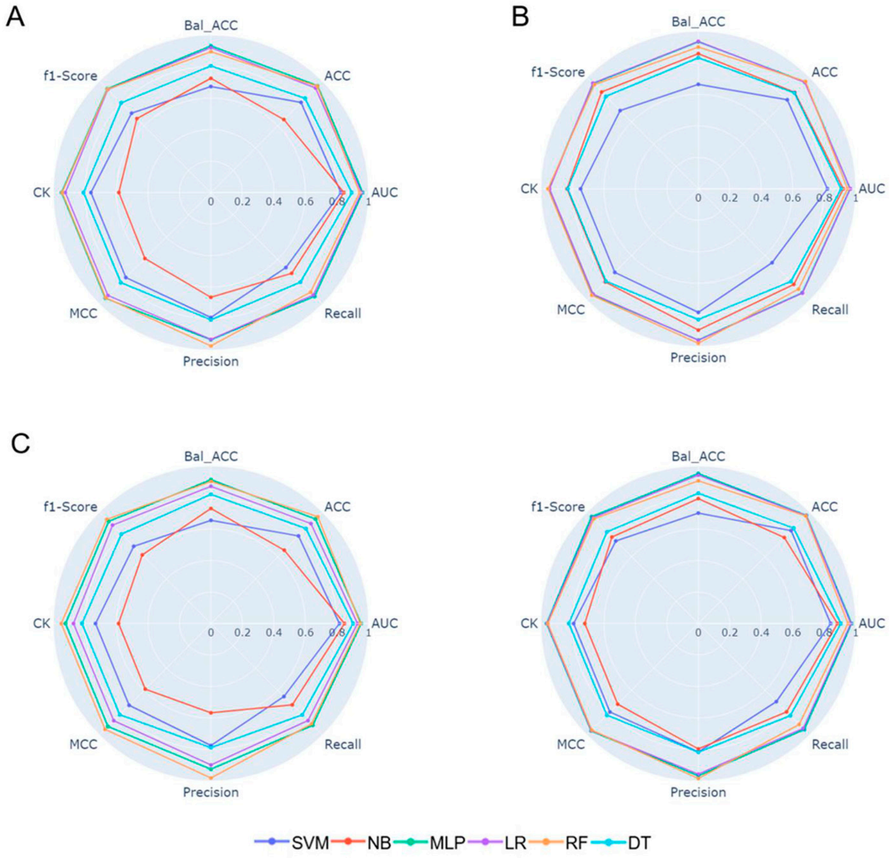

Figure S2. Radar chart of validation sets on Atom-pair fingerprint & molecular descriptors (**A**), ECFP6 fingerprint & molecular descriptors (**B**), MACCS fingerprint & molecular descriptors (**C**), and Atom-pair & ECFP6 & MACCS fingerprints & molecular descriptors (**D**).

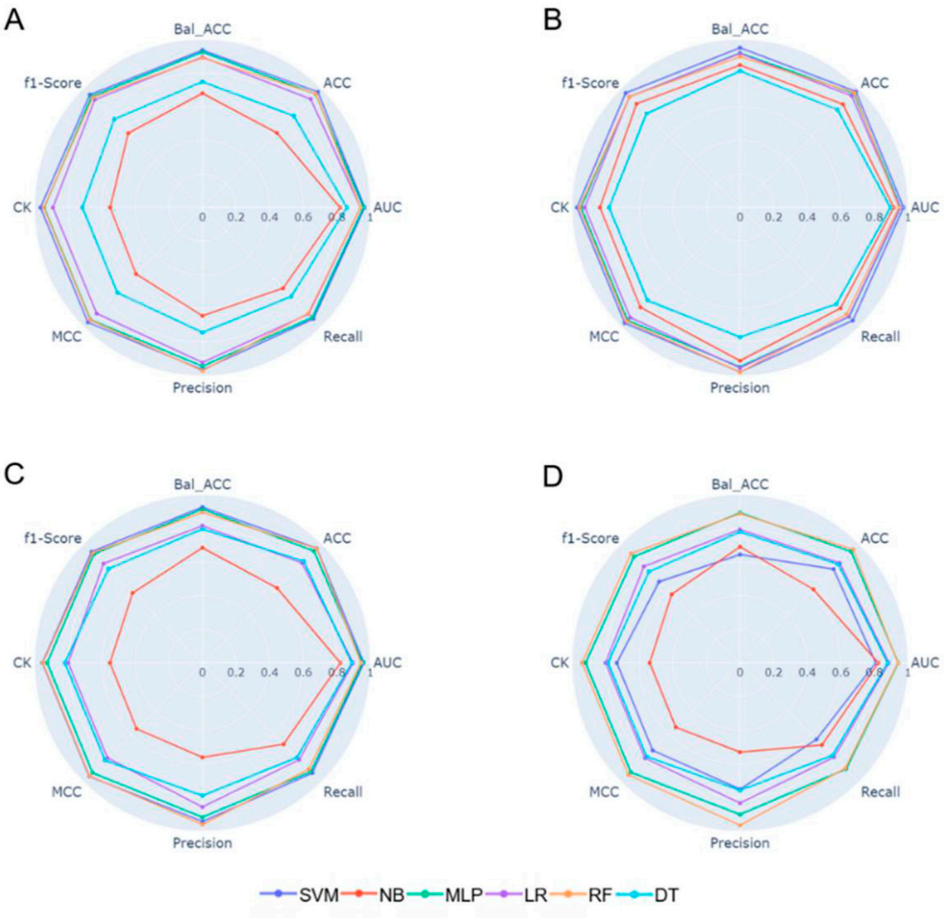

Figure S3. Radar chart of test sets on Atom-pair (A), ECFP6 (B), MACCS fingerprints (C), and molecular descriptors (D).

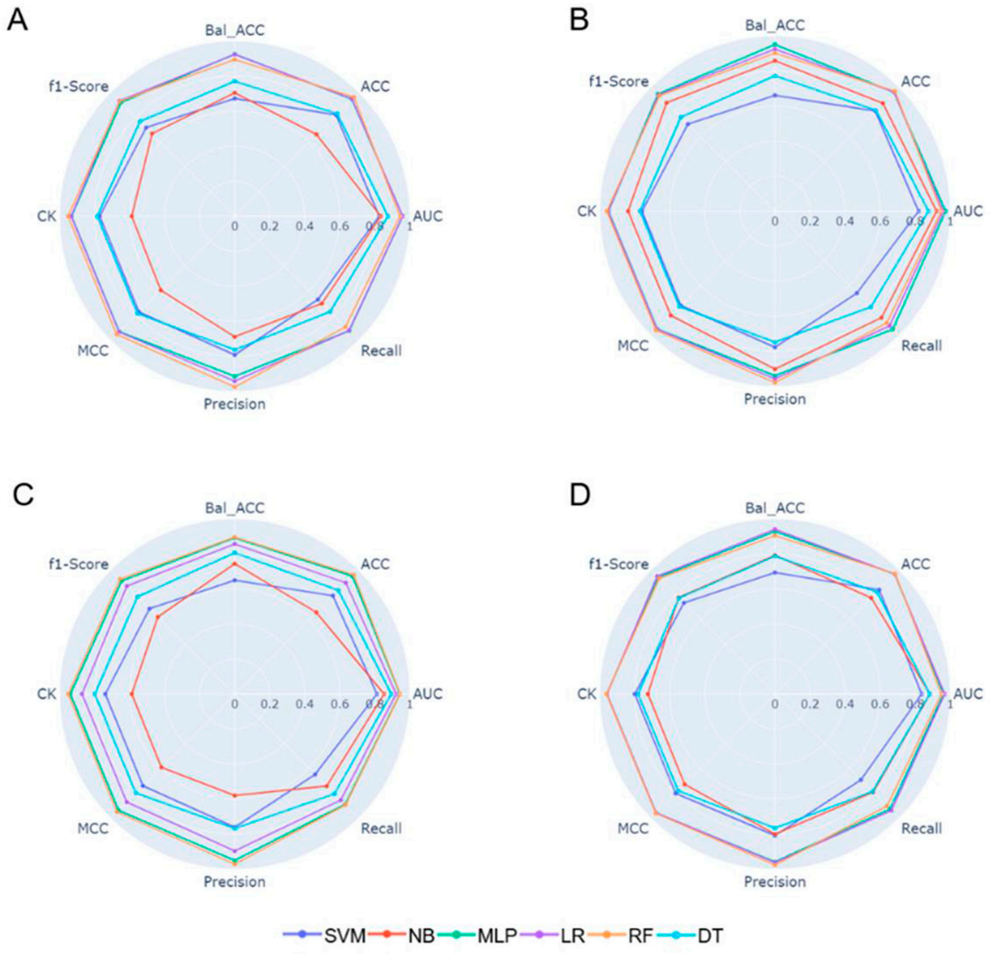

Figure S4. Radar chart of test sets on Atom-pair fingerprint & Molecular Descriptors (**A**), ECFP6 fingerprint & Molecular Descriptors (**B**), MACCS fingerprint & Molecular Descriptors (**C**), and Atom-pair & ECFP6 & MACCS fingerprints & Molecular Descriptors (**D**).

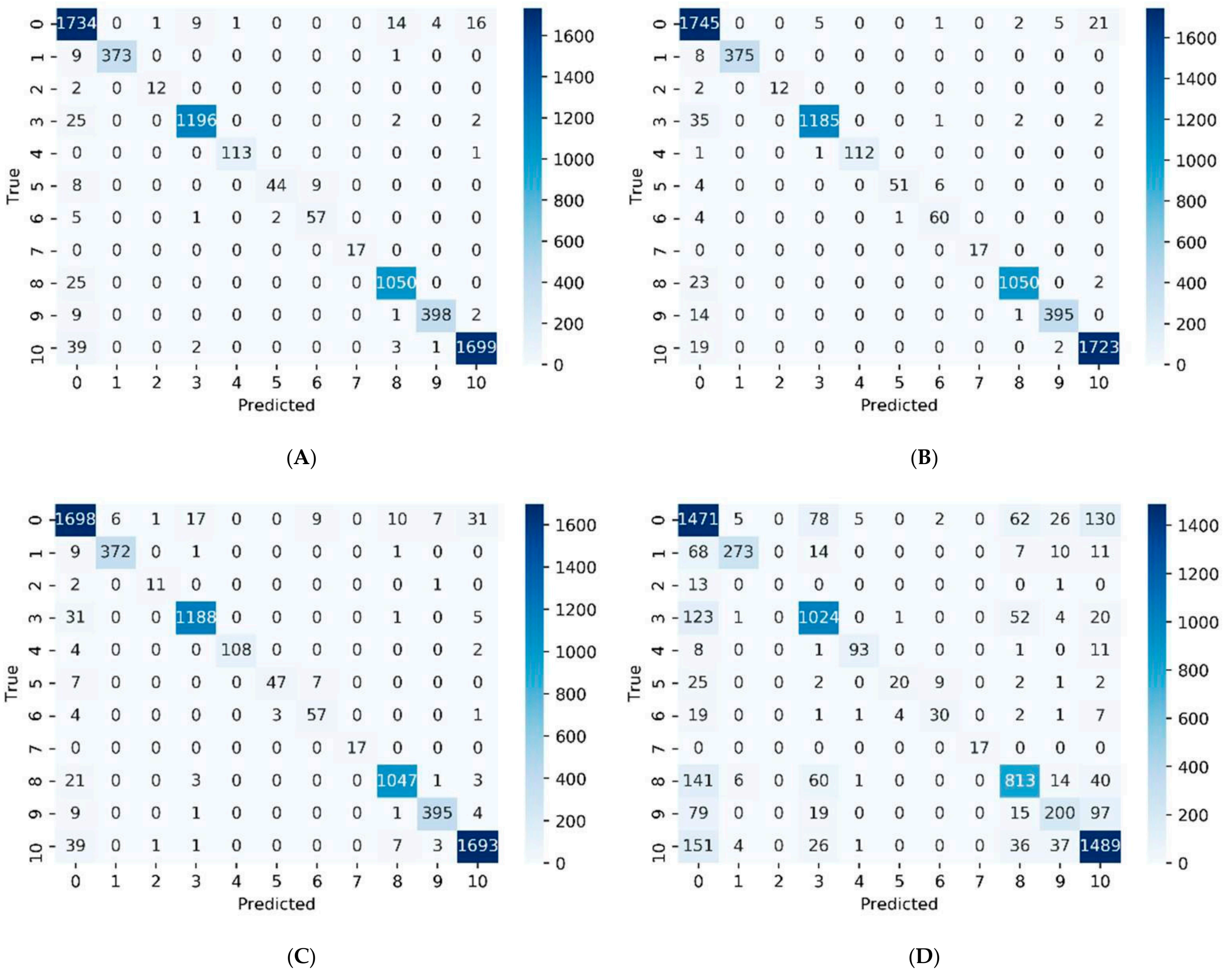

Figure S5. Confusion matrix (m) representing SVM classification performance on test set constructed with Atom-pair fingerprints (**A**), ECFP6 fingerprints (**B**), MACCS fingerprints (**C**), and molecular descriptors (**D**). The element m(i, j) is the number of times an observation of the ith true class was predicted to be of the jth class. Each colored cell of the confusion matrix chart corresponds to one element of the confusion matrix. Drug-like molecules, CB1, FFA2, mAchR M1, S1P3, GLP1-R, GCGR, PTHrP, mGlu2, mGlu4, and mGlu5 were labeled as 0 to 10, respectively.

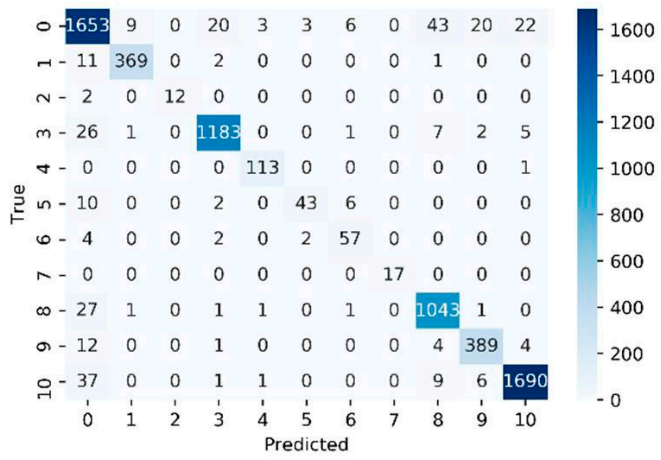

(**A**)

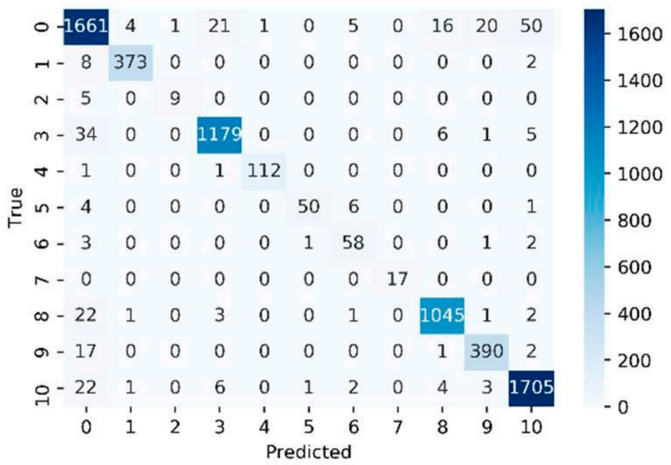

(**B**)

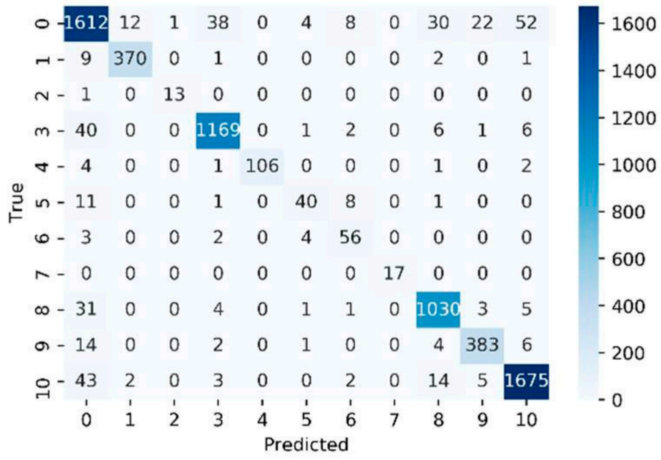

(**C**)

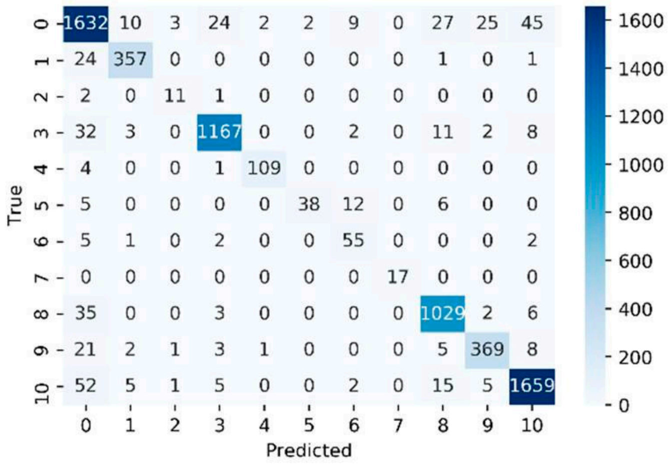

(**D**)

Figure S6. Confusion matrix (m) representing MLP classification performance on test set constructed with Atom-pair fingerprints (**A**), ECFP6 fingerprints (**B**), MACCS fingerprints (**C**), and molecular descriptors (**D**). The element m(i, j) is the number of times an observation of the ith true class was predicted to be of the jth class. Each colored cell of the confusion matrix chart corresponds to one element of the confusion matrix. Drug-like molecules, CB1, FFA2, mAchR M1, S1P3, GLP1-R, GCGR, PTHrP, mGlu2, mGlu4, and mGlu5 were labeled as 0 to 10, respectively.

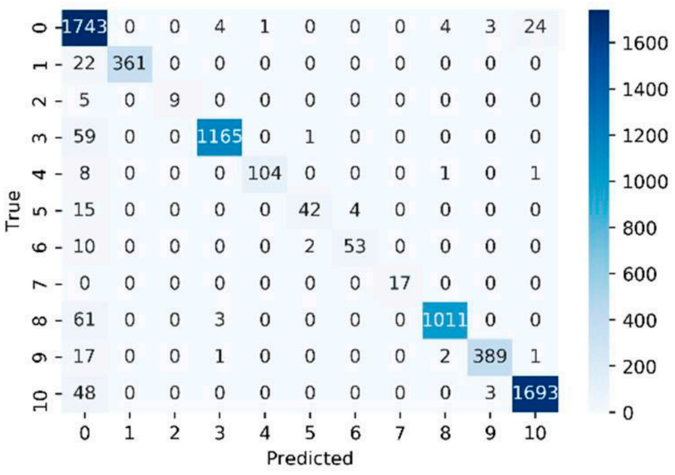

(**A**)

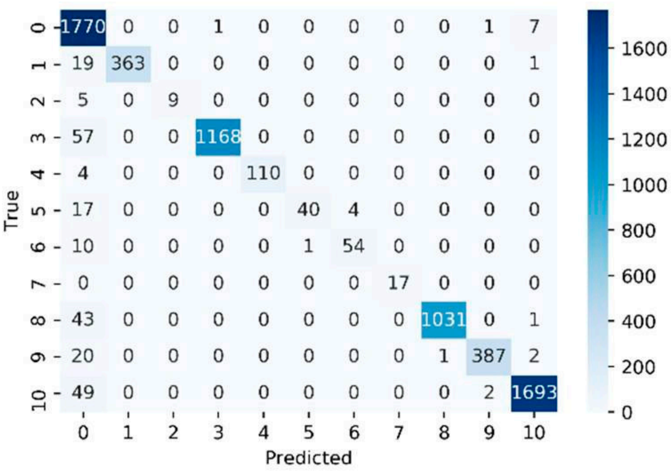

(**B**)

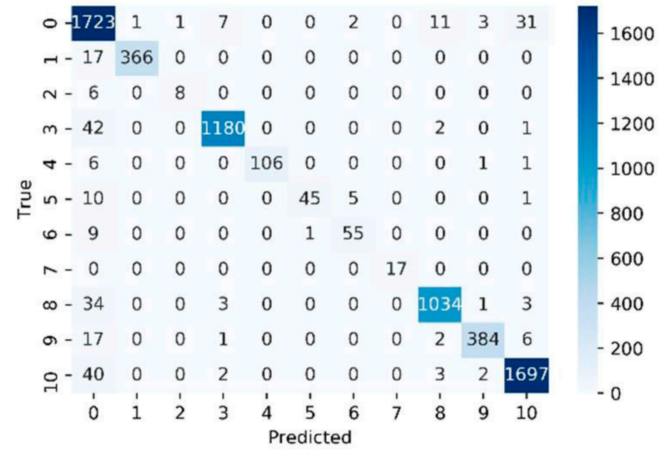

(**C**)

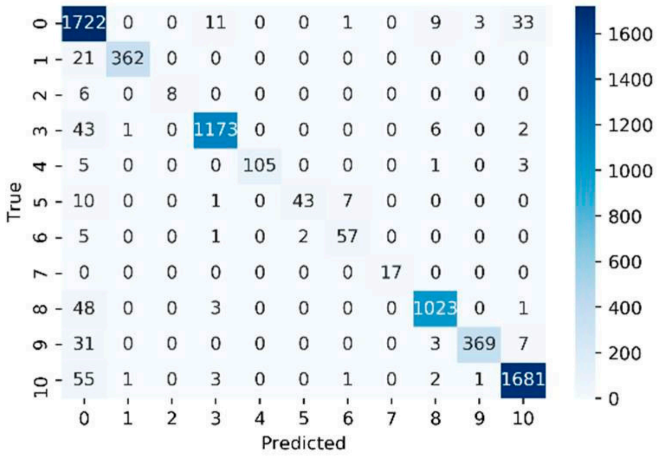

(**D**)

Figure S7. Confusion matrix (m) representing RF classification performance on test set constructed with Atom-pair fingerprints (**A**), ECFP6 fingerprints (**B**), MACCS fingerprints (**C**), and molecular descriptors (**D**). The element m(i, j) is the number of times an observation of the ith true class was predicted to be of the jth class. Each colored cell of the confusion matrix chart corresponds to one element of the confusion matrix. Drug-like molecules, CB1, FFA2, mAchR M1, S1P3, GLP1-R, GCGR, PTHrP, mGlu2, mGlu4, and mGlu5 were labeled as 0 to 10, respectively.

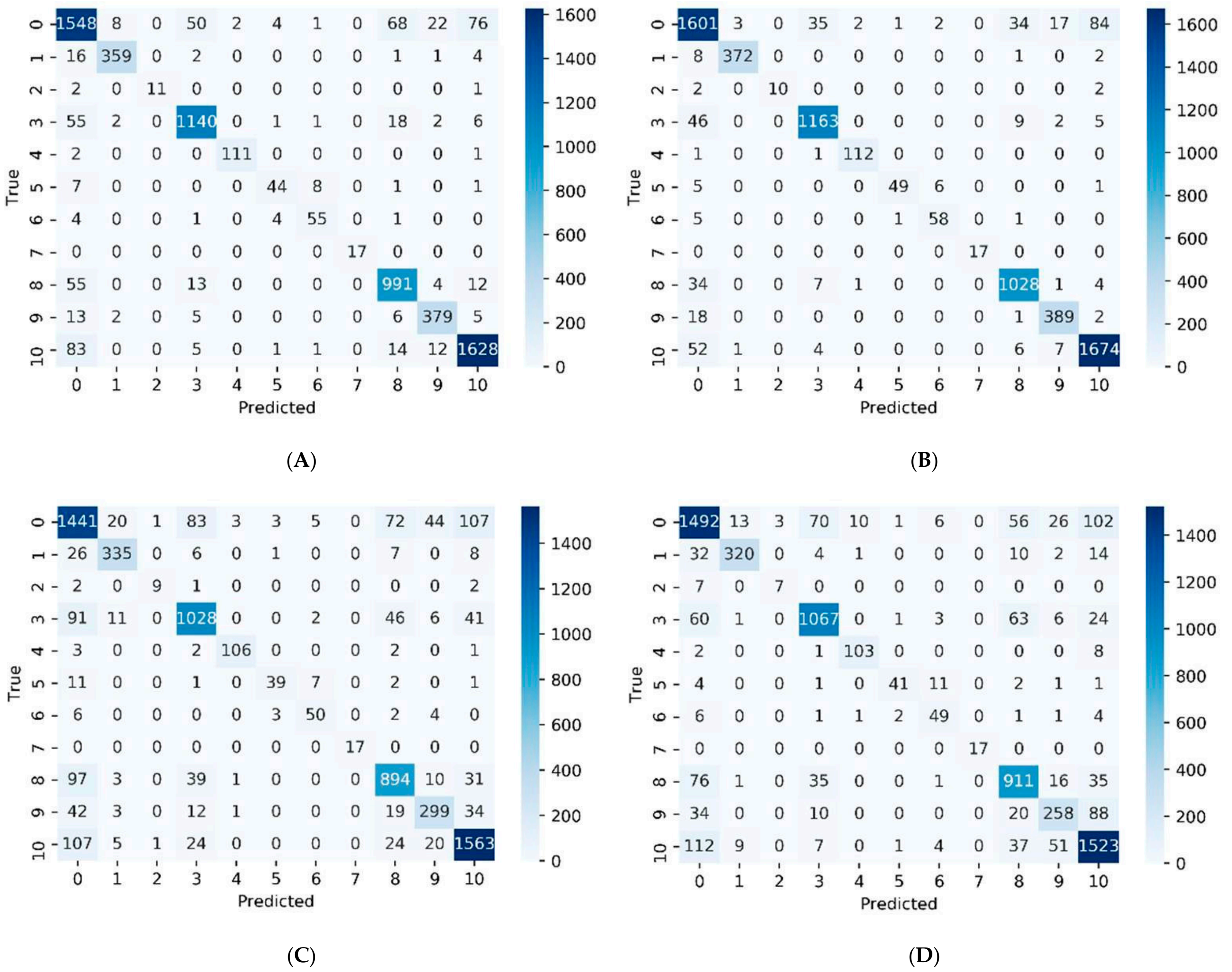

Figure S8. Confusion matrix (m) representing LR classification performance on test set constructed with Atom-pair fingerprints (**A**), ECFP6 fingerprints (**B**), MACCS fingerprints (**C**), and molecular descriptors (**D**). The element m(i, j) is the number of times an observation of the ith true class was predicted to be of the jth class. Each colored cell of the confusion matrix chart corresponds to one element of the confusion matrix. Drug-like molecules, CB1, FFA2, mAchR M1, S1P3, GLP1-R, GCGR, PTHrP, mGlu2, mGlu4, and mGlu5 were labeled as 0 to 10, respectively.

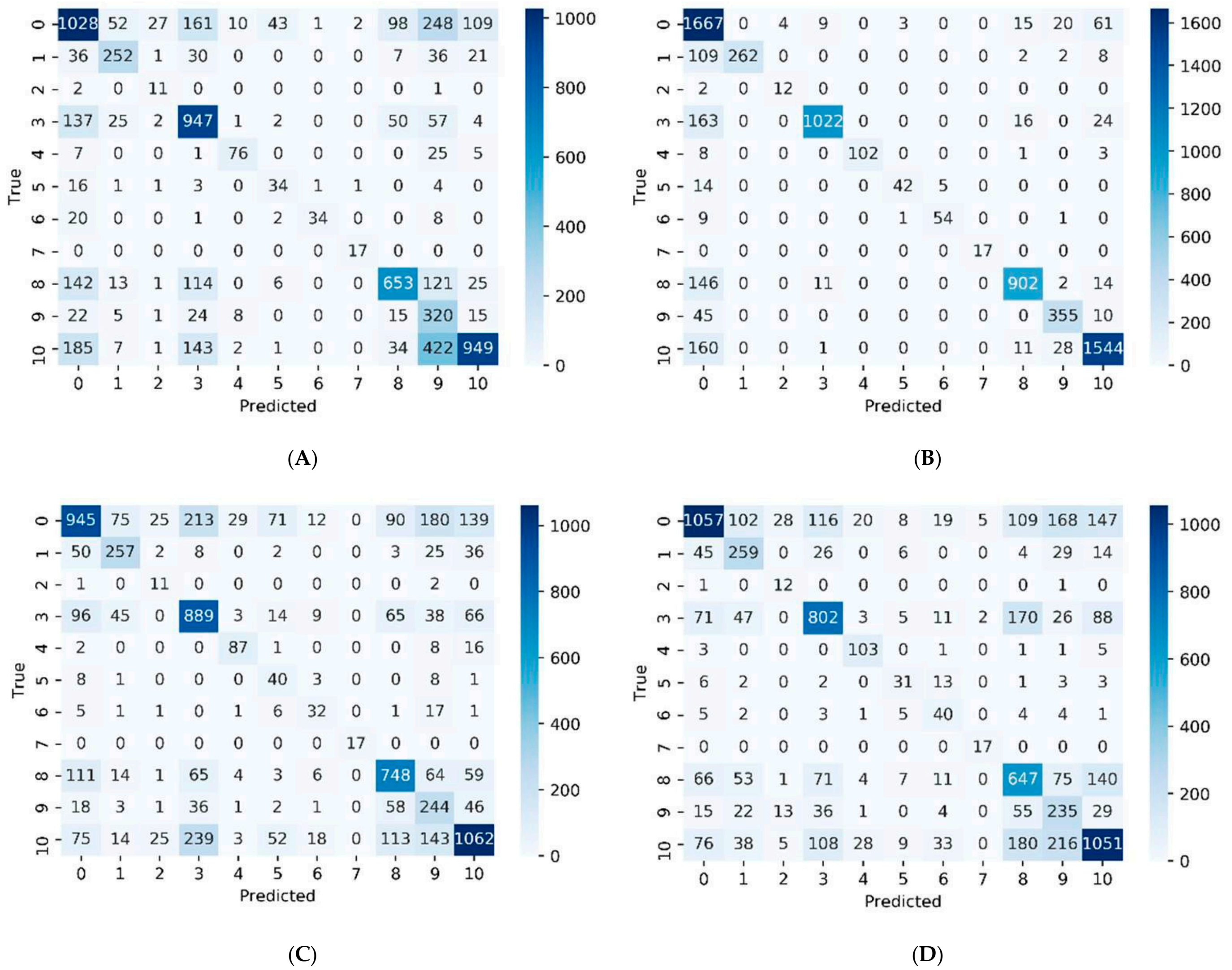

Figure S9. Confusion matrix (m) representing NB classification performance on test set constructed with Atom-pair fingerprints (**A**), ECFP6 fingerprints (**B**), MACCS fingerprints (**C**), and molecular descriptors (**D**). The element m(i, j) is the number of times an observation of the ith true class was predicted to be of the jth class. Each colored cell of the confusion matrix chart corresponds to one element of the confusion matrix. Drug-like molecules, CB1, FFA2, mAchR M1, S1P3, GLP1-R, GCGR, PTHrP, mGlu2, mGlu4, and mGlu5 were labeled as 0 to 10, respectively.

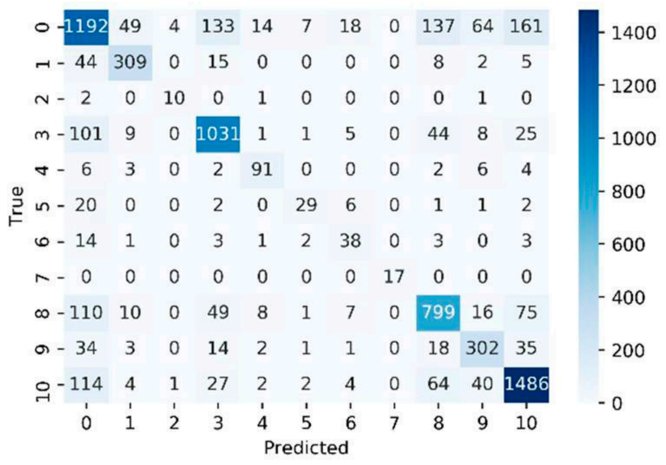

(**A**)

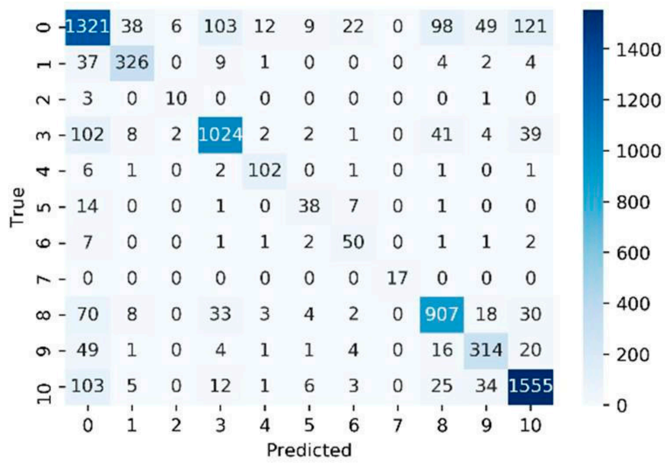

(**B**)

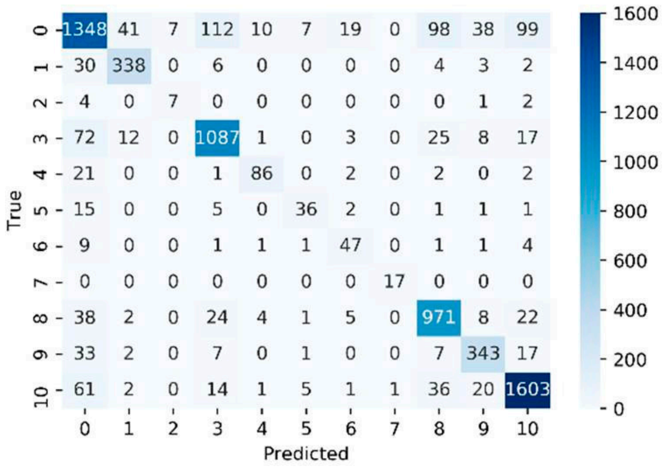

(**C**)

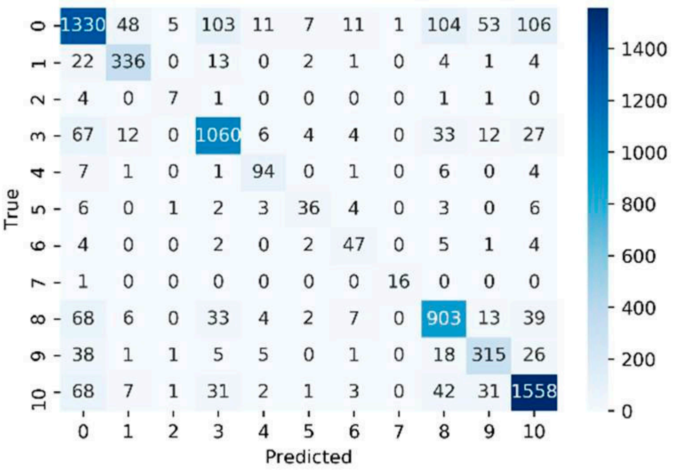

(**D**)

Figure S10. Confusion matrix (m) representing DT classification performance on test set constructed with Atom-pair fingerprints (**A**), ECFP6 fingerprints (**B**), MACCS fingerprints (**C**), and molecular descriptors (**D**). The element m(i, j) is the number of times an observation of the ith true class was predicted to be of the jth class. Each colored cell of the confusion matrix chart corresponds to one element of the confusion matrix. Drug-like molecules, CB1, FFA2, mAchR M1, S1P3, GLP1-R, GCGR, PTHrP, mGlu2, mGlu4, and mGlu5 were labeled as 0 to 10, respectively.

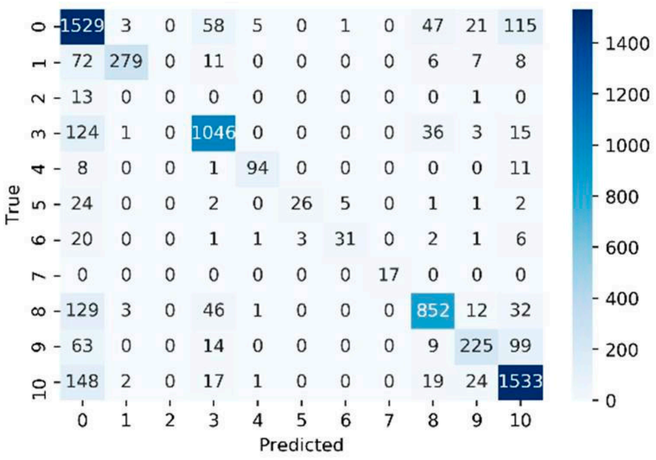

(**A**)

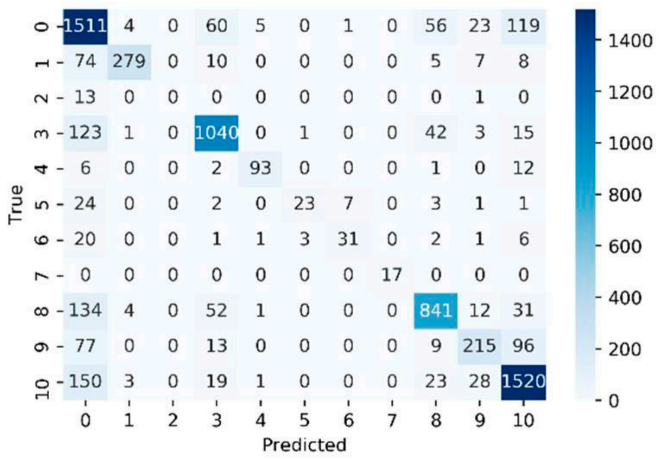

(**B**)

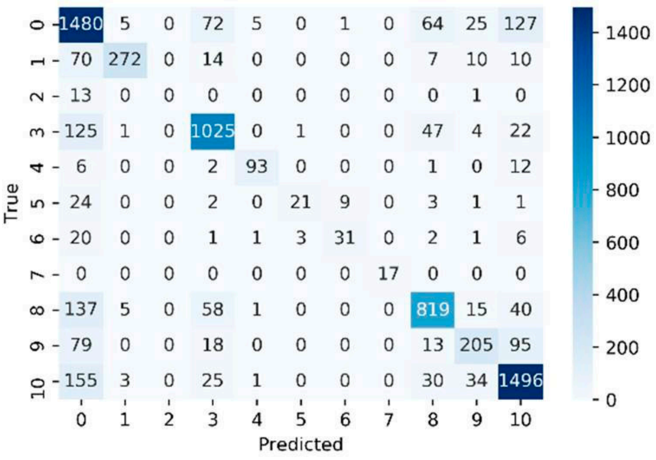

(**C**)

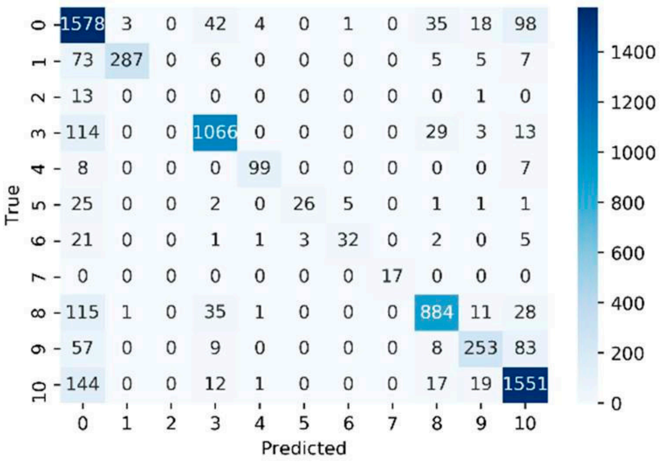

(**D**)

Figure S11. Confusion matrix (m) representing SVM classification performance on test set constructed with Atom-pair & molecular descriptors (**A**), ECFP6 & molecular descriptors (**B**), MACCS & molecular descriptors (**C**), and Atom-pair & ECFP6 & MACCS & molecular descriptors (**D**). The element m(i, j) is the number of times an observation of the ith true class was predicted to be of the jth class. Each colored cell of the confusion matrix chart corresponds to one element of the confusion matrix. Drug-like molecules, CB1, FFA2, mAchR M1, S1P3, GLP1-R, GCGR, PTHrP, mGlu2, mGlu4, and mGlu5 were labeled as 0 to 10, respectively.

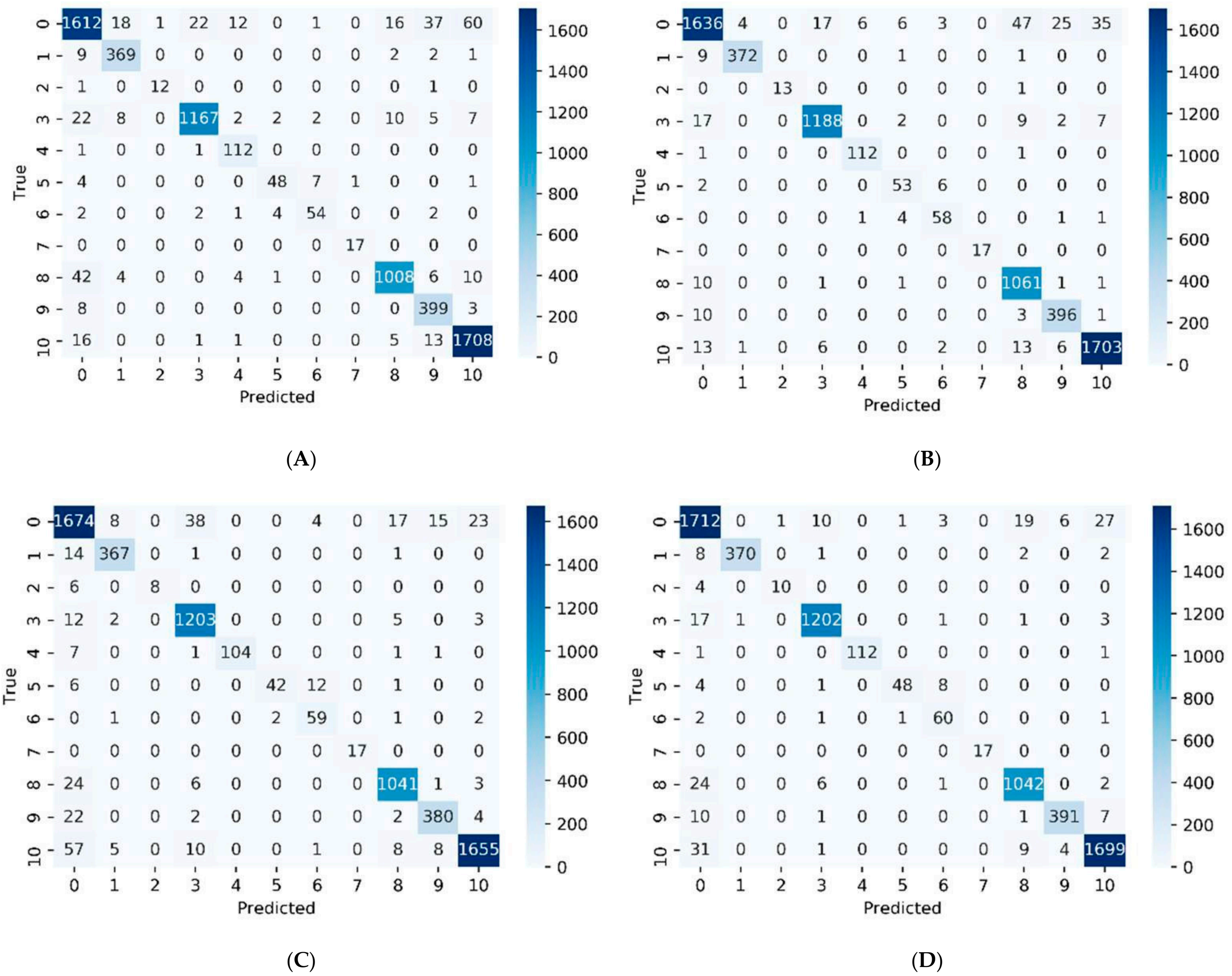

Figure S12. Confusion matrix (m) representing MLP classification performance on test set constructed with Atom-pair & molecular descriptors (**A**), ECFP6 & molecular descriptors (**B**), MACCS & molecular descriptors (**C**), and Atom-pair & ECFP6 & MACCS & molecular descriptors (**D**). The element m(i, j) is the number of times an observation of the ith true class was predicted to be of the jth class. Each colored cell of the confusion matrix chart corresponds to one element of the confusion matrix. Drug-like molecules, CB1, FFA2, mAchR M1, S1P3, GLP1-R, GCGR, PTHrP, mGlu2, mGlu4, and mGlu5 were labeled as 0 to 10, respectively.

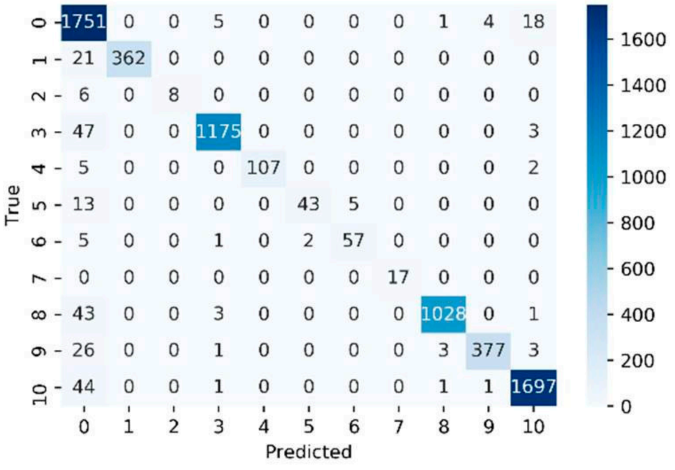

(**A**)

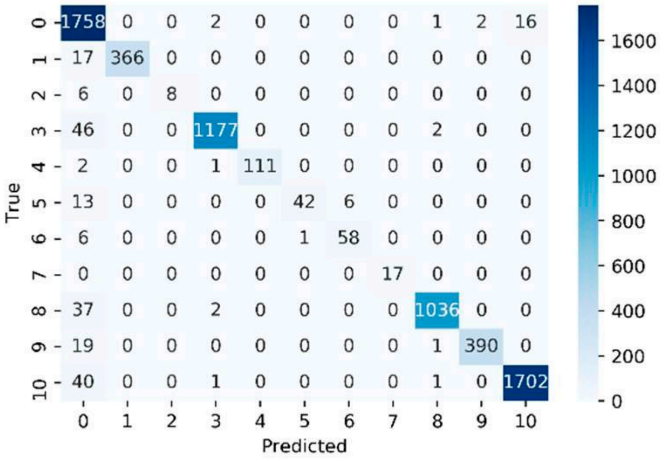

(**B**)

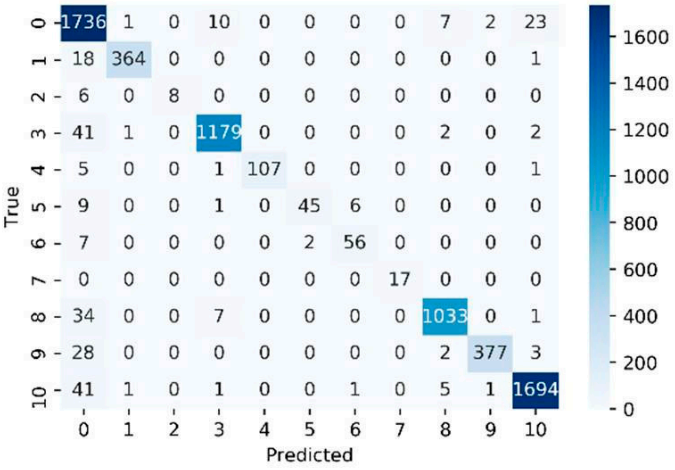

(**C**)

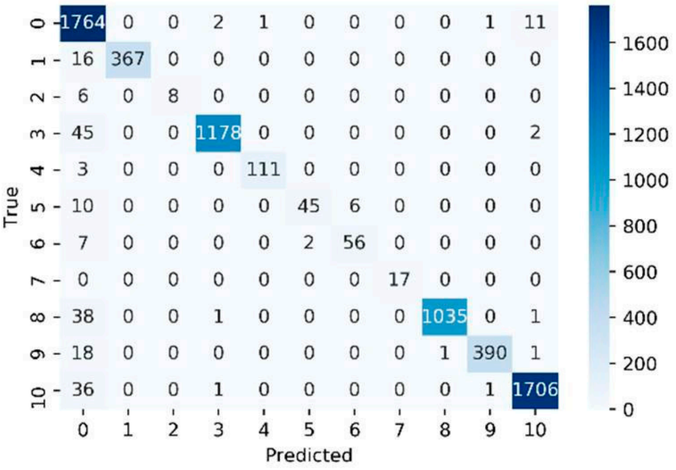

(**D**)

Figure S13. Confusion matrix (m) representing RF classification performance on test set constructed with Atom-pair & molecular descriptors (**A**), ECFP6 & molecular descriptors (**B**), MACCS & molecular descriptors (**C**), and Atom-pair & ECFP6 & MACCS & molecular descriptors (**D**). The element m(i, j) is the number of times an observation of the ith true class was predicted to be of the jth class. Each colored cell of the confusion matrix chart corresponds to one element of the confusion matrix. Drug-like molecules, CB1, FFA2, mAchR M1, S1P3, GLP1-R, GCGR, PTHrP, mGlu2, mGlu4, and mGlu5 were labeled as 0 to 10, respectively.

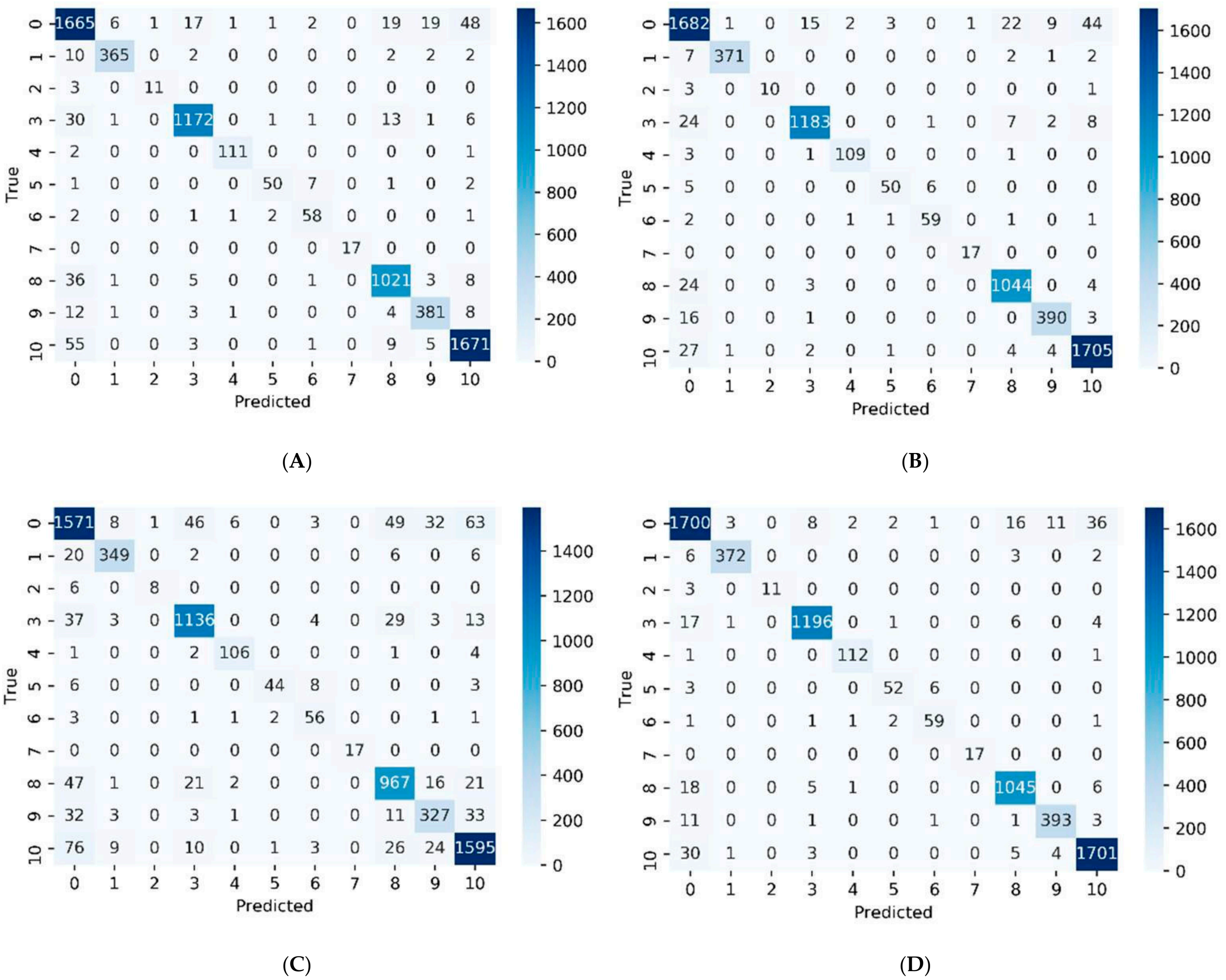

Figure S14. Confusion matrix (m) representing LR classification performance on test set constructed with Atom-pair & molecular descriptors (**A**), ECFP6 & molecular descriptors (**B**), MACCS & molecular descriptors (**C**), and Atom-pair & ECFP6 & MACCS & molecular descriptors (**D**). The element m(i, j) is the number of times an observation of the ith true class was predicted to be of the jth class. Each colored cell of the confusion matrix chart corresponds to one element of the confusion matrix. Drug-like molecules, CB1, FFA2, mAchR M1, S1P3, GLP1-R, GCGR, PTHrP, mGlu2, mGlu4, and mGlu5 were labeled as 0 to 10, respectively.

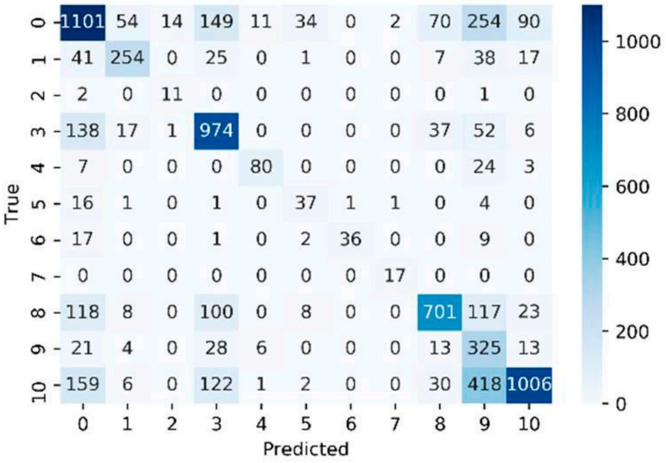

(**A**)

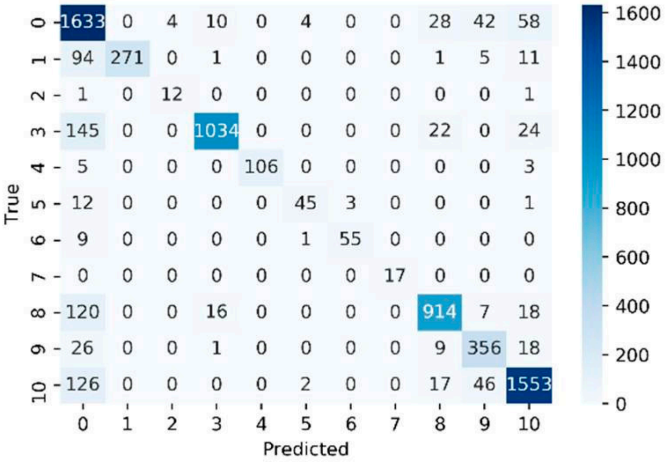

(**B**)

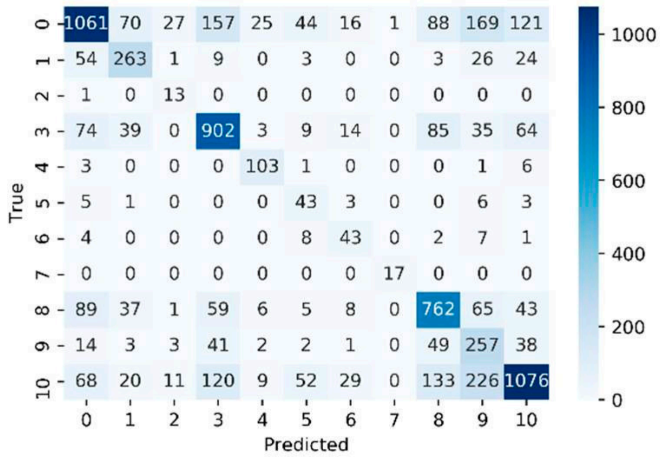

(**C**)

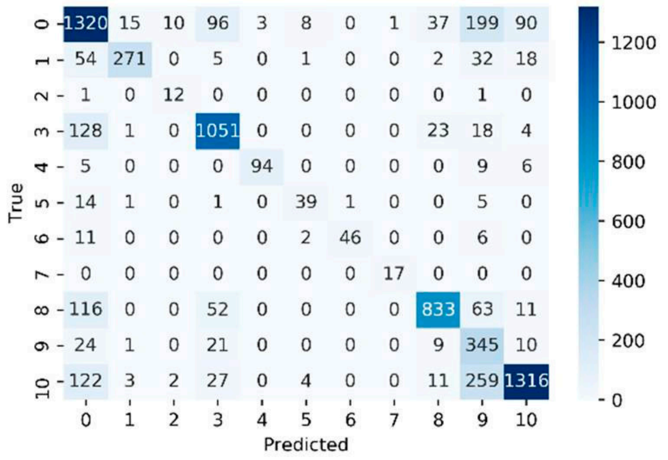

(**D**)

Figure S15. Confusion matrix (m) representing NB classification performance on test set constructed with Atom-pair & molecular descriptors (A), ECFP6 & molecular descriptors (B), MACCS & molecular descriptors (C), and Atom-pair & ECFP6 & MACCS & molecular descriptors (D). The element m(i, j) is the number of times an observation of the ith true class was predicted to be of the jth class. Each colored cell of the confusion matrix chart corresponds to one element of the confusion matrix. Drug-like molecules, CB1, FFA2, mAchR M1, S1P3, GLP1-R, GCGR, PTHrP, mGlu2, mGlu4, and mGlu5 were labeled as 0 to 10, respectively.

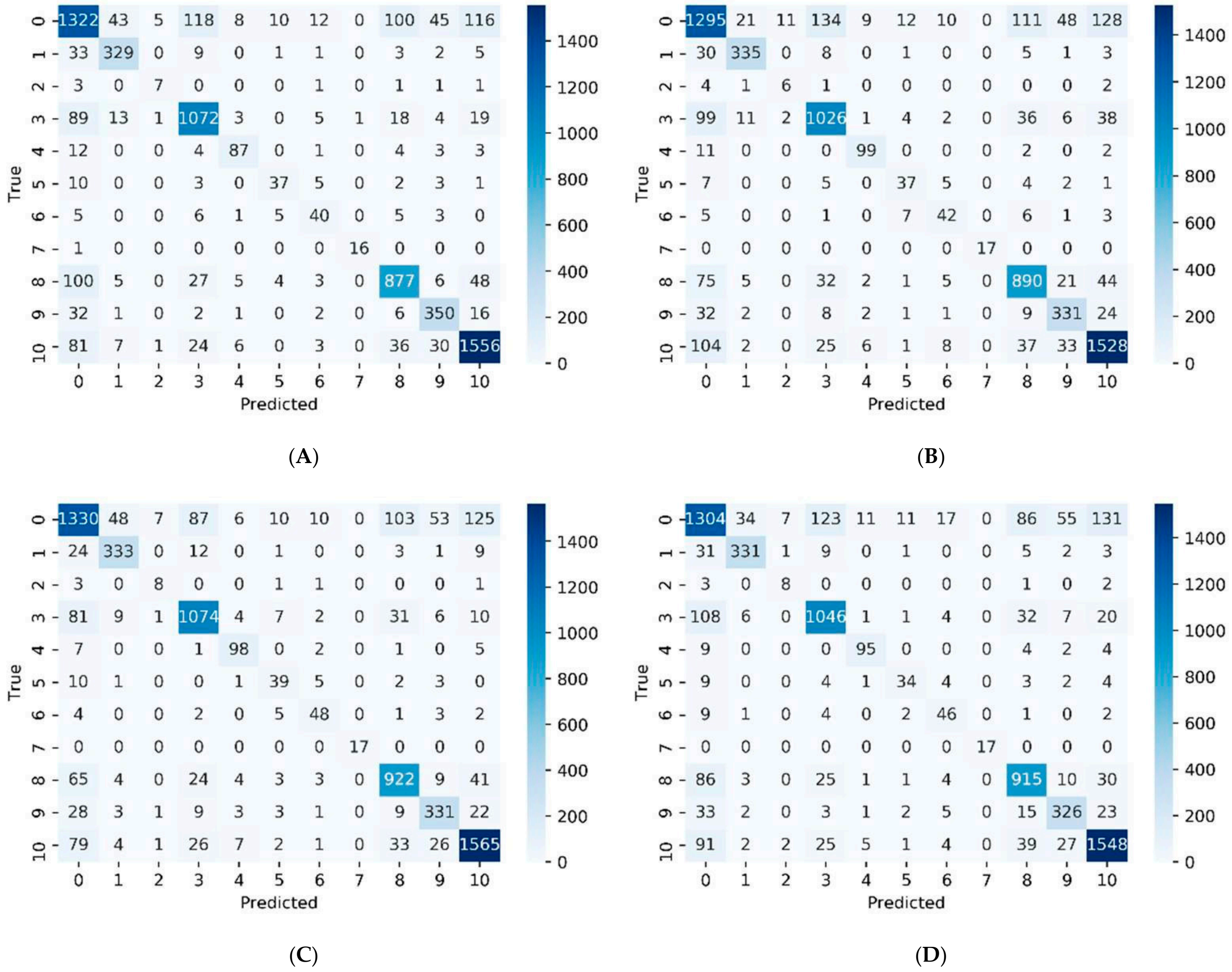

Figure S16. Confusion matrix (m) representing DT classification performance on test set constructed with Atom-pair & molecular descriptors (**A**), ECFP6 & molecular descriptors (**B**), MACCS & molecular descriptors (**C**), and Atom-pair & ECFP6 & MACCS & molecular descriptors (**D**). The element m(i, j) is the number of times an observation of the ith true class was predicted to be of the jth class. Each colored cell of the confusion matrix chart corresponds to one element of the confusion matrix. Drug-like molecules, CB1, FFA2, mAchR M1, S1P3, GLP1-R, GCGR, PTHrP, mGlu2, mGlu4, and mGlu5 were labeled as 0 to 10, respectively.

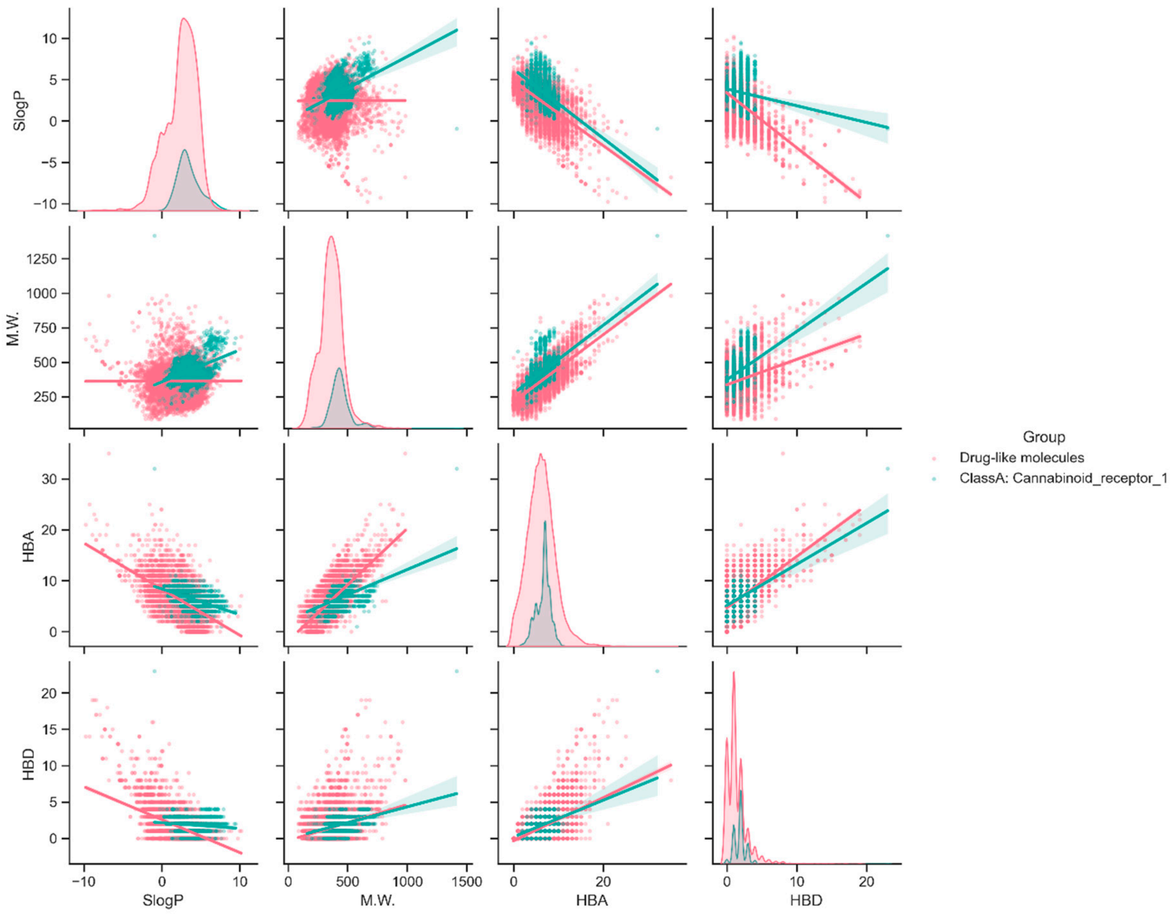

Figure S17. Pair-wise distribution comparison of CB1 and drug-like molecules on SlogP, molecular weight (M.W.), hydrogen bond acceptor (HBA), and hydrogen bond doner (HBD).

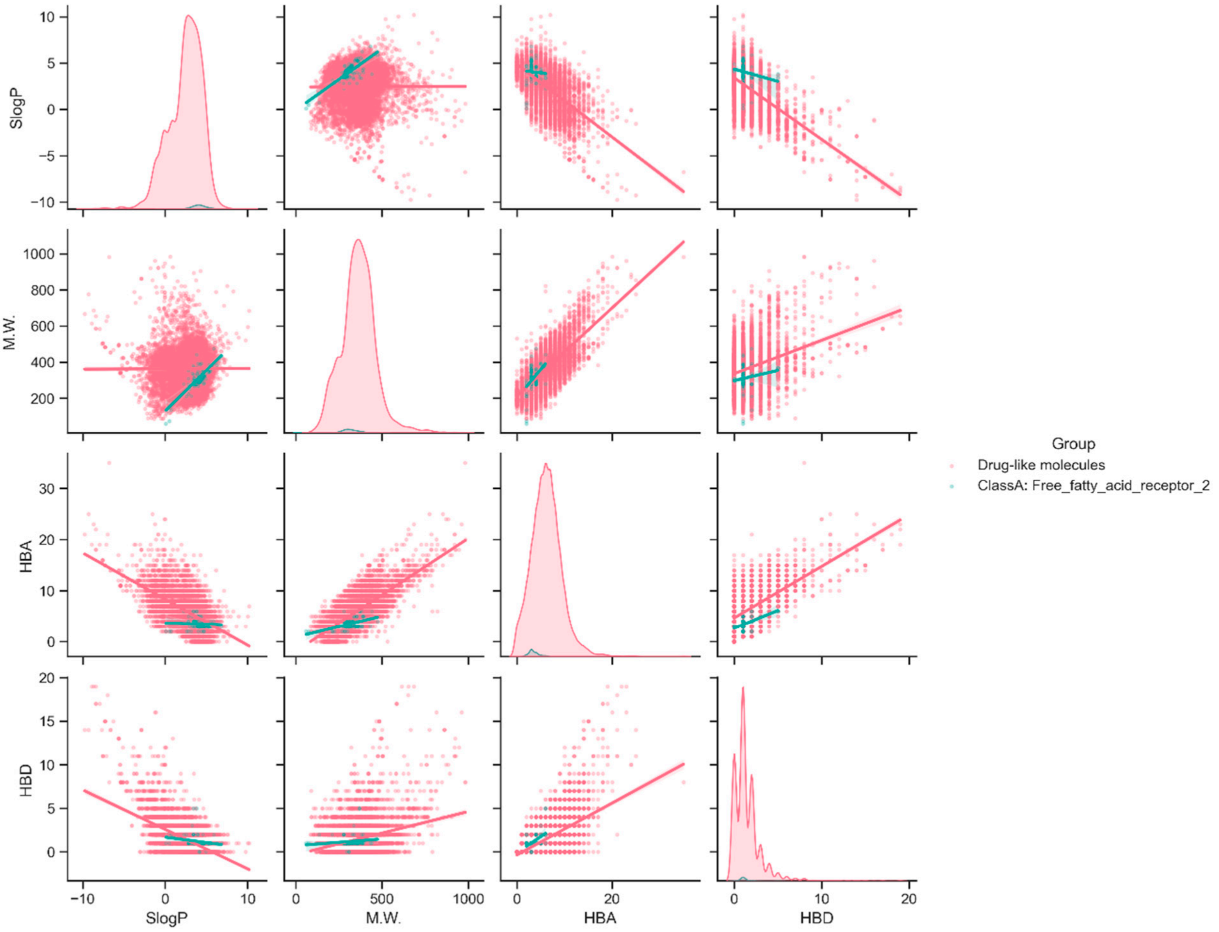

Figure S18. Pair-wise distribution comparison of FFA2 and drug-like molecules on SlogP, molecular weight (M.W.), hydrogen bond acceptor (HBA), and hydrogen bond doner (HBD).

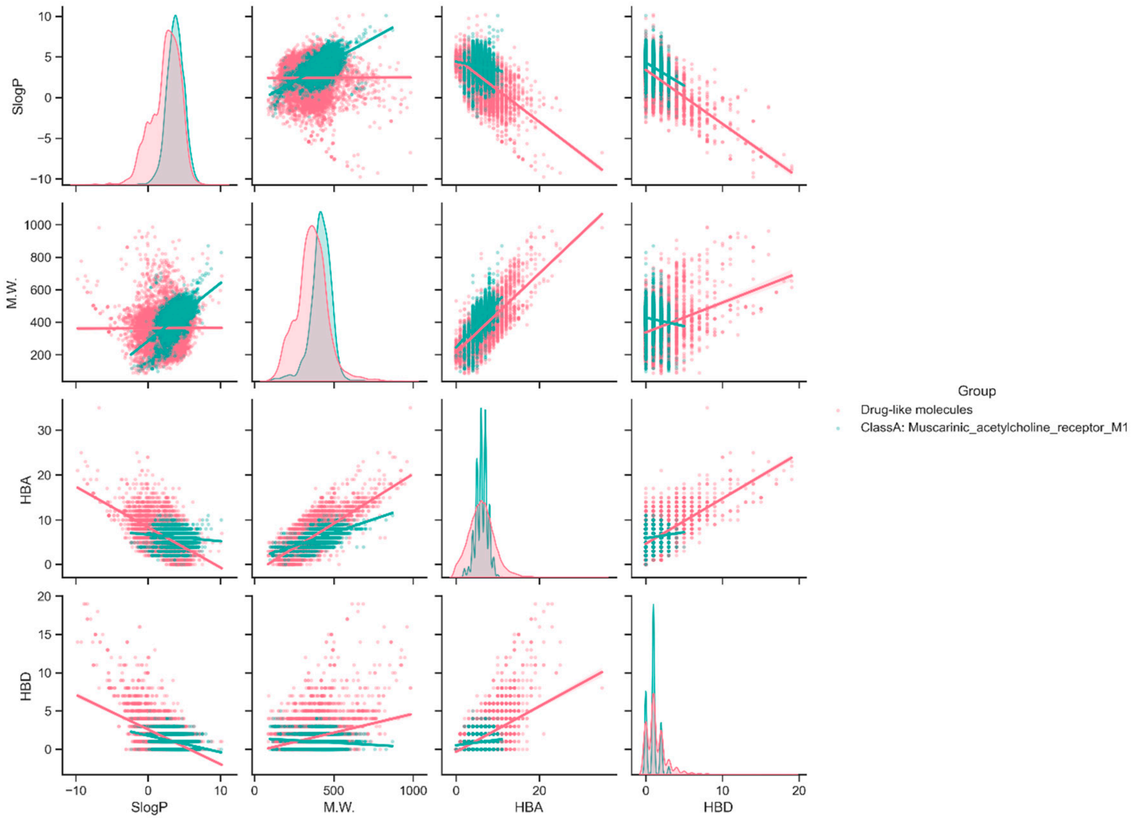

Figure S19. Pair-wise distribution comparison of mAchR M1 and drug-like molecules on SlogP, molecular weight (M.W.), hydrogen bond acceptor (HBA), and hydrogen bond doner (HBD).

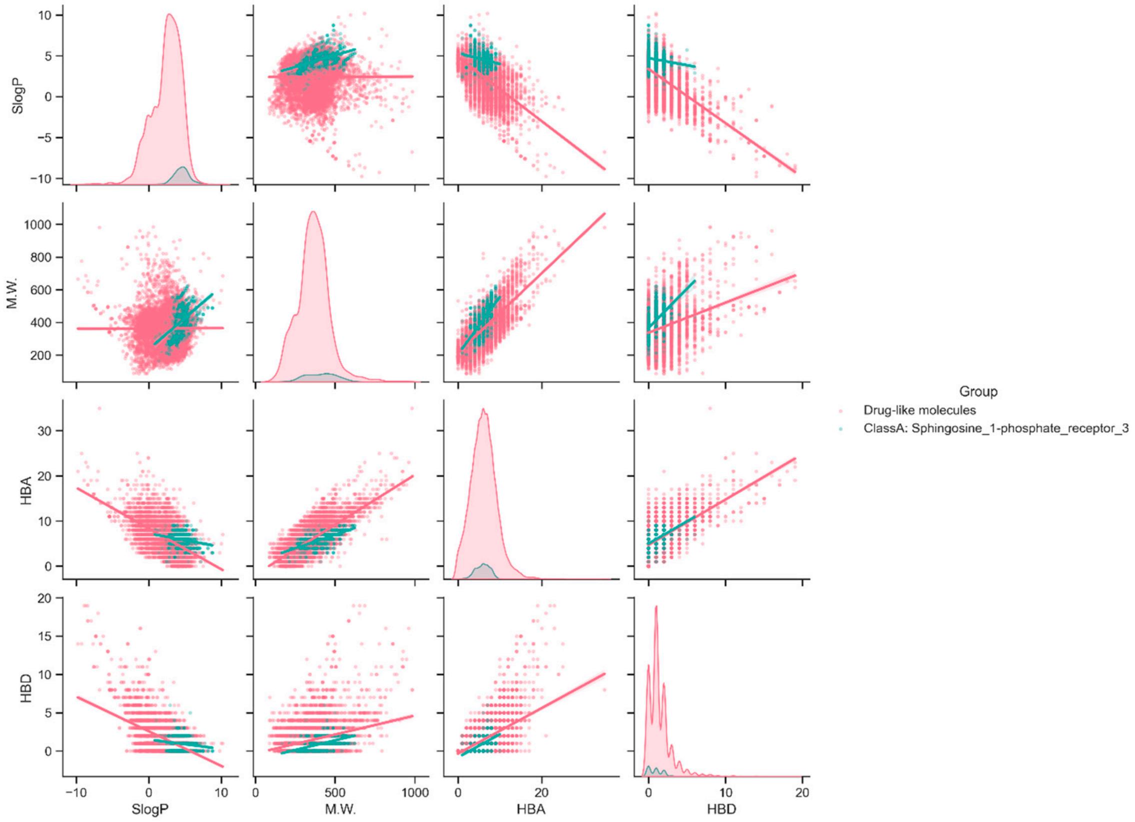

Figure S20. Pair-wise distribution comparison of S1P3 and drug-like molecules on SlogP, molecular weight (M.W.), hydrogen bond acceptor (HBA), and hydrogen bond doner (HBD).

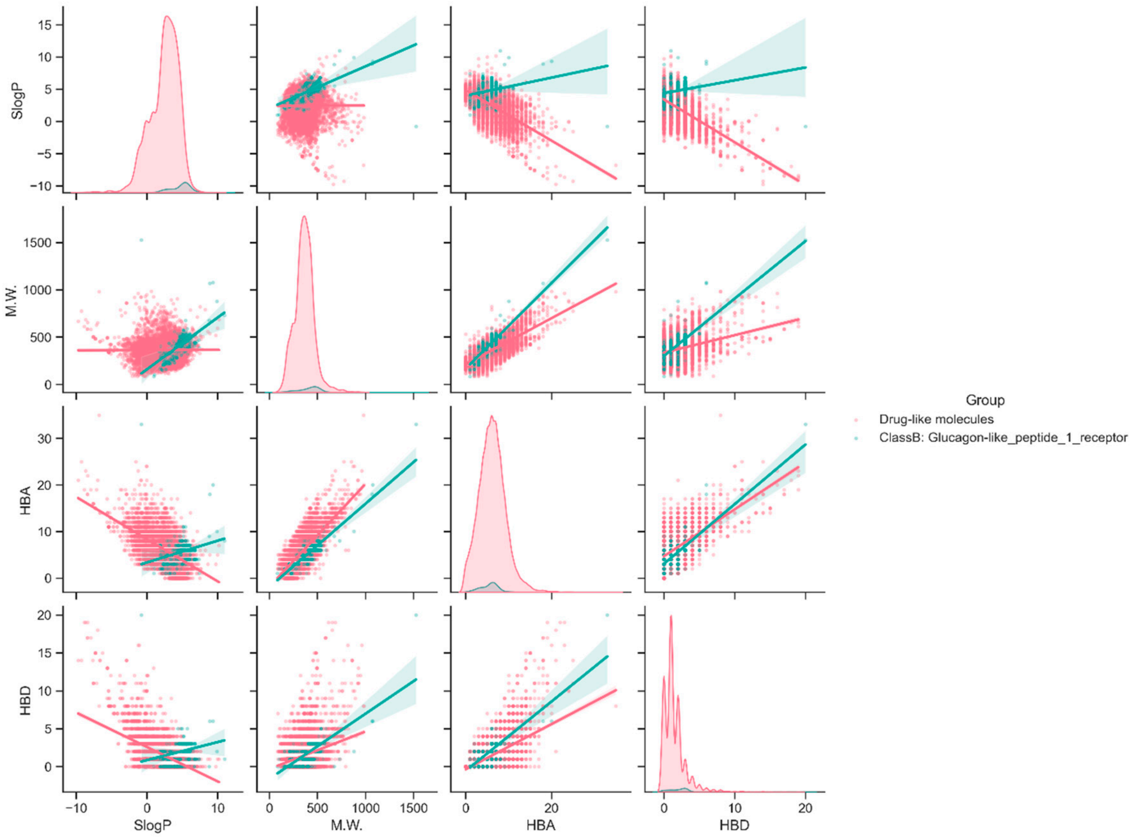

Figure S21. Pair-wise distribution comparison of GLP1-R and drug-like molecules on SlogP, molecular weight (M.W.), hydrogen bond acceptor (HBA), and hydrogen bond doner (HBD).

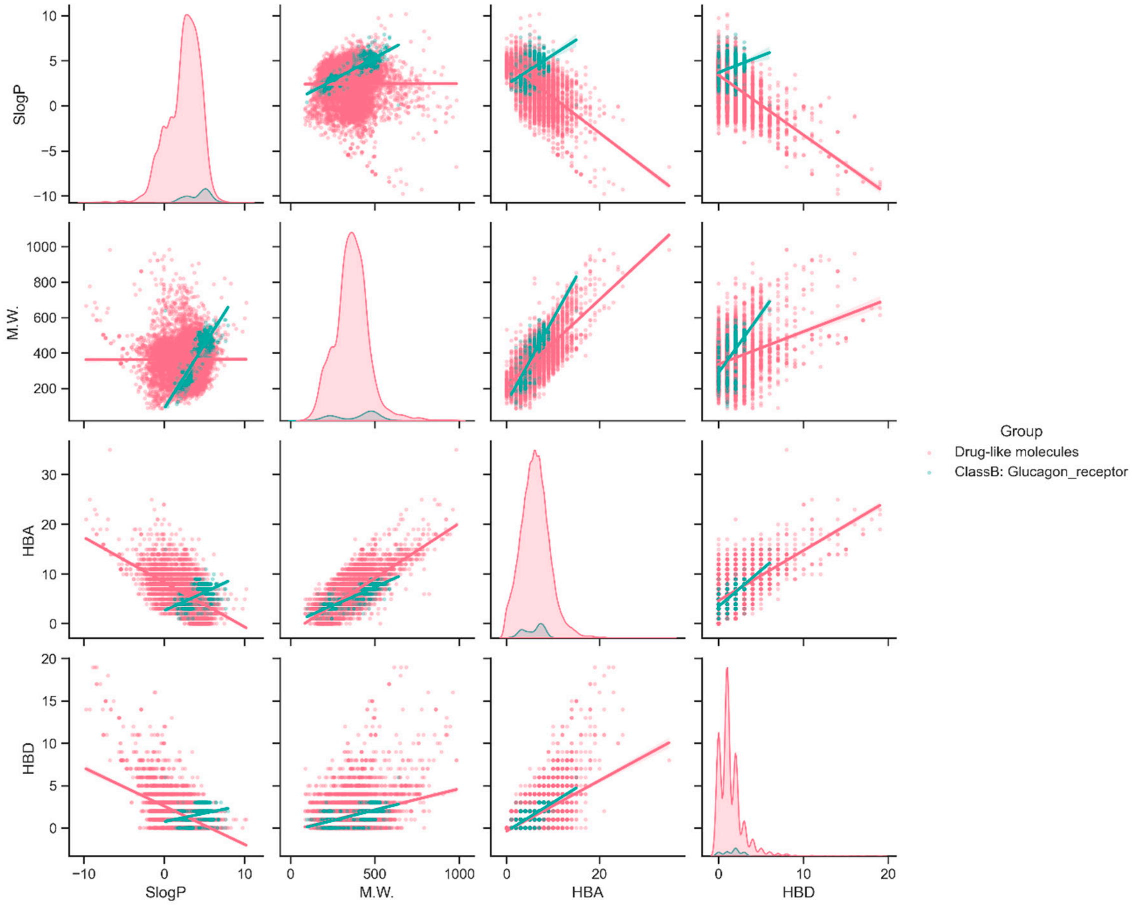

Figure S22. Pair-wise distribution comparison of GCGR and drug-like molecules on SlogP, molecular weight (M.W.), hydrogen bond acceptor (HBA), and hydrogen bond doner (HBD).

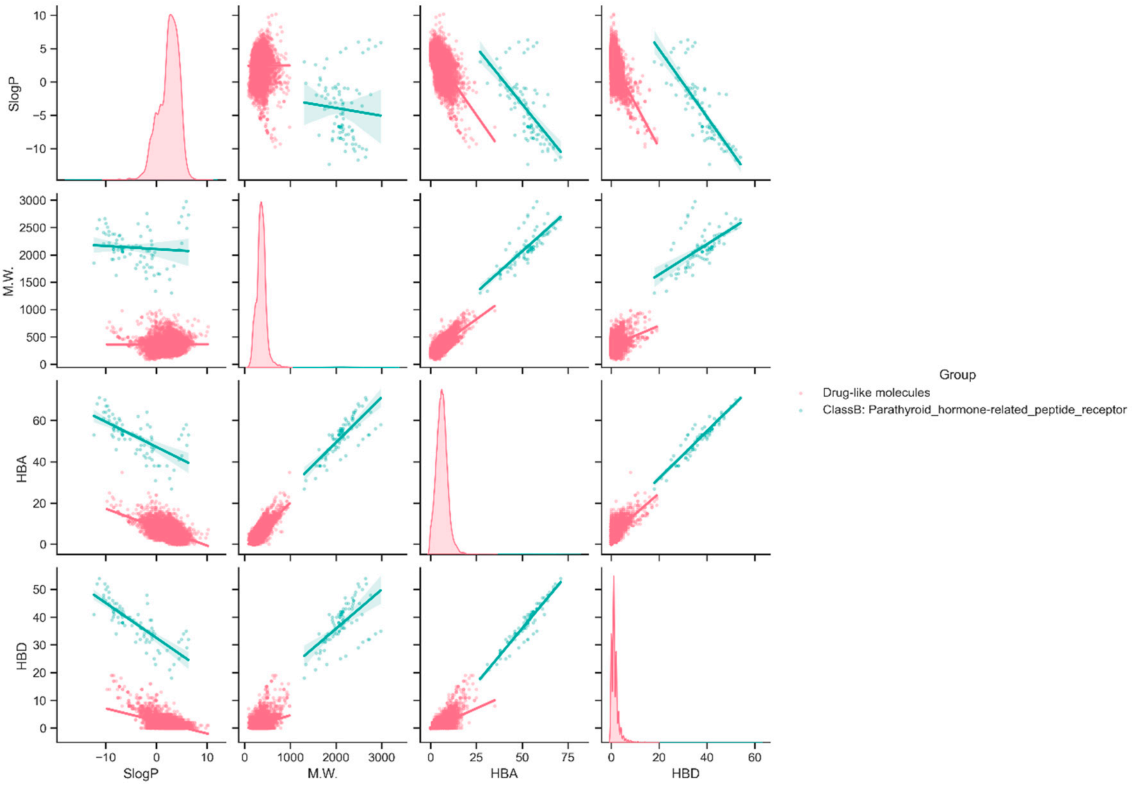

Figure S23. Pair-wise distribution comparison of PTHrP and drug-like molecules on SlogP, molecular weight (M.W.), hydrogen bond acceptor (HBA), and hydrogen bond doner (HBD).

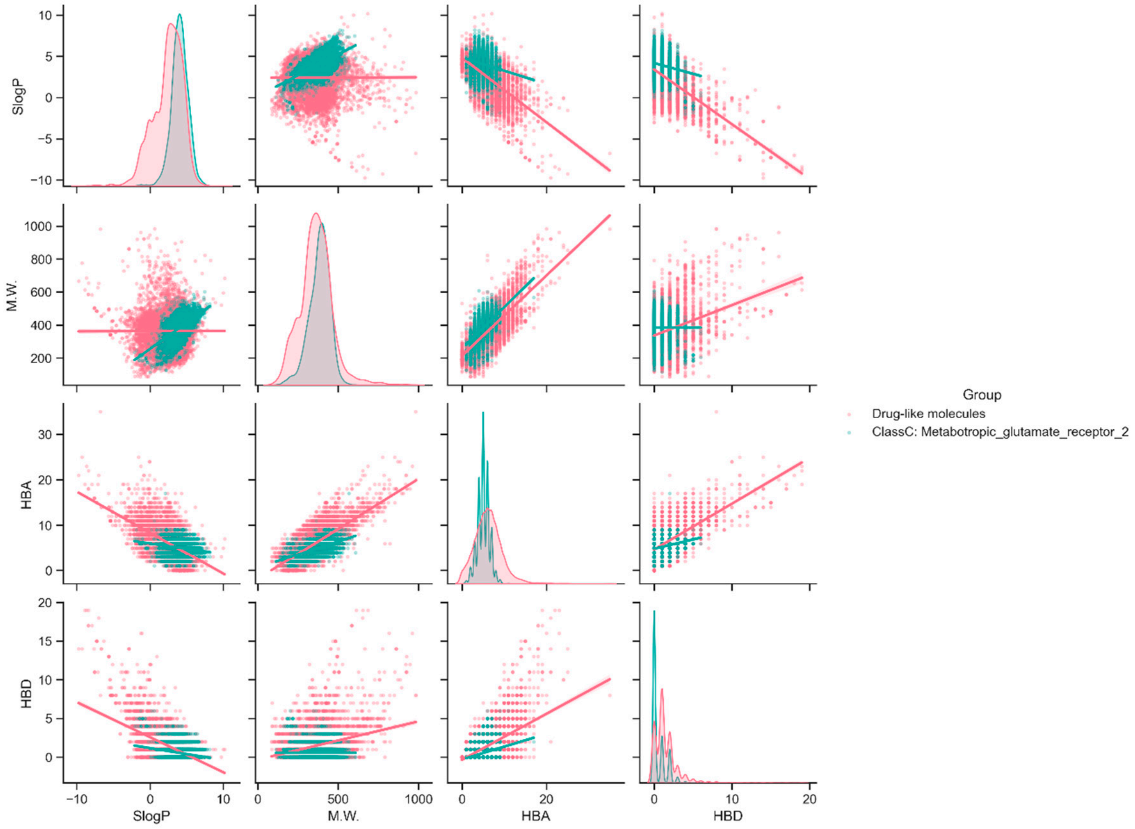

Figure S24. Pair-wise distribution comparison of mGlu2 and drug-like molecules on SlogP, molecular weight (M.W.), hydrogen bond acceptor (HBA), and hydrogen bond doner (HBD).

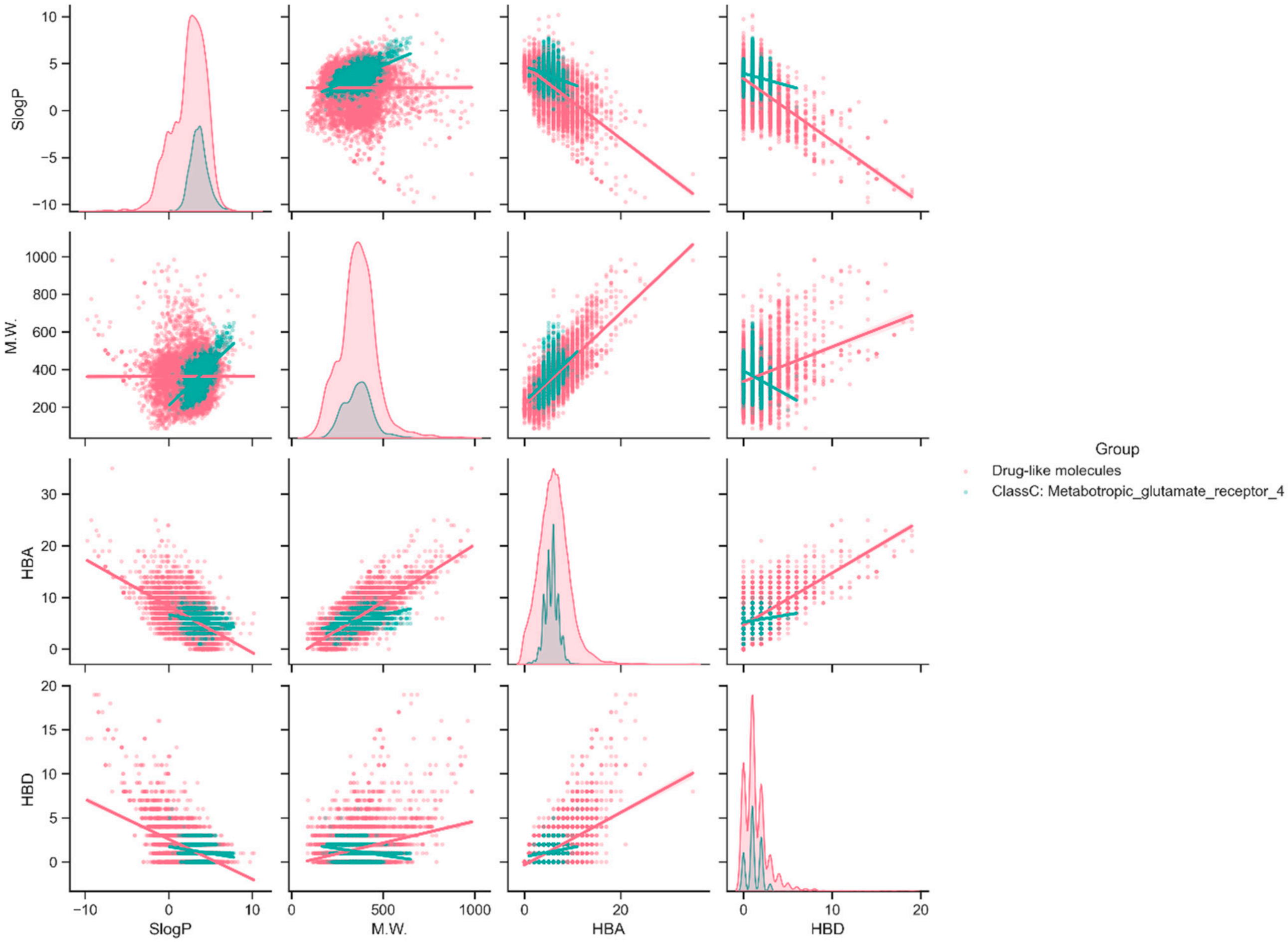

Figure S25. Pair-wise distribution comparison of mGlu4 and drug-like molecules on SlogP, molecular weight (M.W.), hydrogen bond acceptor (HBA), and hydrogen bond doner (HBD).

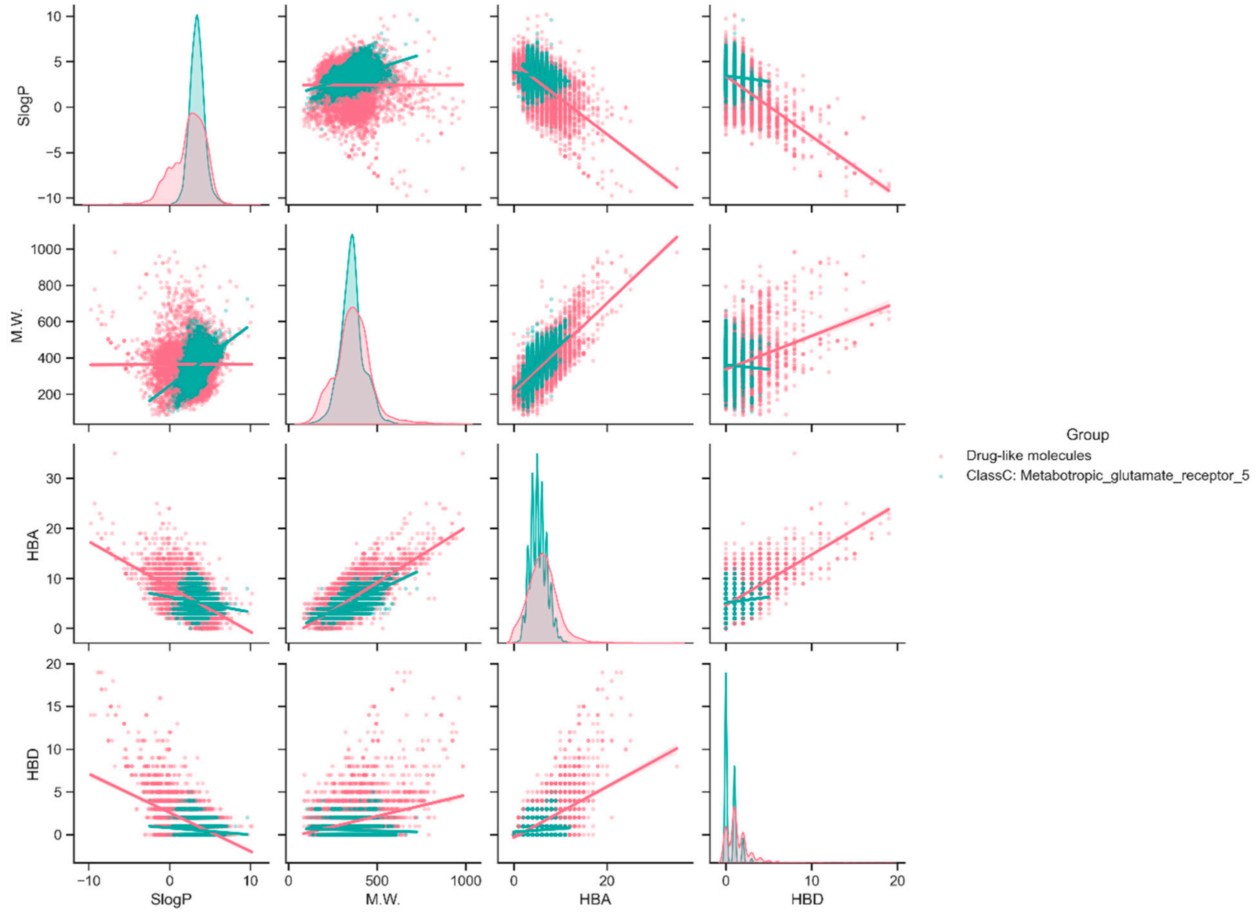

Figure S26. Pair-wise distribution comparison of mGlu5 and drug-like molecules on SlogP, molecular weight (M.W.), hydrogen bond acceptor (HBA), and hydrogen bond doner (HBD).

Table S1. Results of the Validation Set on Each Dataset with One Feature Type

Datasets	Model	AUC	ACC	Bal_ACC	$f1$-score	CK	MCC	Precision	Recall
Atom-pair	SVM	0.973	0.969	0.951	0.961	0.961	0.961	0.972	0.951
	NB	0.833	0.623	0.707	0.624	0.546	0.554	0.628	0.707
	MLP	0.949	0.945	0.904	0.920	0.932	0.932	0.939	0.904
	LR	0.940	0.903	0.890	0.905	0.880	0.880	0.921	0.890
	RF	0.940	0.949	0.886	0.923	0.937	0.938	0.973	0.886
	DT	0.849	0.785	0.722	0.730	0.735	0.735	0.744	0.722
ECFP6	SVM	0.977	0.977	0.957	0.967	0.972	0.972	0.977	0.957
	NB	0.915	0.863	0.847	0.874	0.829	0.833	0.920	0.847
	MLP	0.972	0.960	0.949	0.952	0.950	0.950	0.956	0.949
	LR	0.958	0.944	0.922	0.938	0.930	0.930	0.956	0.922
	RF	0.961	0.962	0.927	0.951	0.953	0.954	0.980	0.927
	DT	0.904	0.867	0.823	0.832	0.836	0.836	0.843	0.823
MACCS	SVM	0.964	0.961	0.933	0.940	0.952	0.952	0.949	0.933
	NB	0.834	0.632	0.708	0.607	0.555	0.557	0.575	0.708
	MLP	0.956	0.938	0.920	0.922	0.923	0.923	0.925	0.920
	LR	0.897	0.838	0.813	0.837	0.799	0.800	0.871	0.813
	RF	0.945	0.955	0.894	0.924	0.944	0.945	0.962	0.894
	DT	0.908	0.869	0.831	0.823	0.839	0.839	0.816	0.831
molecular descriptors	SVM	0.812	0.781	0.649	0.687	0.725	0.727	0.770	0.649
	NB	0.824	0.616	0.688	0.562	0.537	0.540	0.512	0.688
	MLP	0.941	0.931	0.890	0.898	0.915	0.915	0.909	0.890
	LR	0.890	0.836	0.799	0.811	0.796	0.797	0.830	0.799
	RF	0.949	0.947	0.904	0.932	0.935	0.935	0.969	0.904
	DT	0.893	0.849	0.803	0.806	0.814	0.814	0.811	0.803

Table S2. Results of the Validation Set on Each Dataset with Two Feature Types

Datasets	Model	AUC	ACC	Bal_ACC	$f1$-score	CK	MCC	Precision	Recall
Atom-pair & molecular descriptors	SVM	0.825	0.809	0.673	0.712	0.761	0.763	0.793	0.673
	NB	0.844	0.655	0.725	0.664	0.584	0.592	0.665	0.725
	MLP	0.963	0.958	0.931	0.931	0.948	0.948	0.935	0.931
	LR	0.956	0.939	0.920	0.925	0.924	0.924	0.932	0.920
	RF	0.944	0.955	0.894	0.928	0.944	0.944	0.974	0.894
	DT	0.894	0.845	0.804	0.805	0.808	0.808	0.808	0.804
ECFP6 & molecular descriptors	SVM	0.820	0.799	0.663	0.702	0.748	0.750	0.784	0.663
	NB	0.921	0.866	0.858	0.869	0.834	0.836	0.897	0.858
	MLP	0.964	0.960	0.933	0.944	0.951	0.951	0.960	0.933
	LR	0.966	0.956	0.936	0.948	0.945	0.945	0.962	0.936
	RF	0.948	0.964	0.900	0.934	0.955	0.956	0.981	0.900
	DT	0.908	0.862	0.832	0.830	0.830	0.830	0.830	0.832
MACCS& molecular descriptors	SVM	0.815	0.786	0.665	0.693	0.732	0.734	0.774	0.655
	NB	0.847	0.658	0.730	0.616	0.586	0.589	0.567	0.730
	MLP	0.953	0.938	0.913	0.917	0.923	0.924	0.925	0.913
	LR	0.930	0.897	0.871	0.883	0.872	0.872	0.898	0.871
	RF	0.948	0.959	0.902	0.935	0.950	0.950	0.981	0.902
	DT	0.902	0.852	0.820	0.803	0.818	0.818	0.788	0.820

Table S3. Results of the Validation Set on Dataset with Four Feature Types

Datasets	Model	AUC	ACC	Bal_ACC	$f1$-score	CK	MCC	Precision	Recall
Atom-pair & ECFP6 & MACCS & molecular descriptors	SVM	0.841	0.834	0.701	0.741	0.792	0.794	0.815	0.701
	NB	0.884	0.772	0.793	0.776	0.721	0.724	0.796	0.793
	MLP	0.974	0.971	0.952	0.959	0.964	0.964	0.967	0.952
	LR	0.969	0.969	0.942	0.949	0.961	0.961	0.956	0.942
	RF	0.951	0.965	0.906	0.939	0.956	0.957	0.984	0.906
	DT	0.906	0.856	0.827	0.822	0.822	0.822	0.817	0.827

Table S4. The $f1$-score among Different GPCR Families on Each Dataset (Average over all classes within the Same GPCR Family)

Datasets	Model	GPCR A	GPCR B	GPCR C
Atom-pair	SVM	0.963	0.897	0.980
	NB	0.623	0.683	0.575
	MLP	0.959	0.876	0.957
	LR	0.936	0.868	0.920
	RF	0.919	0.887	0.970
	DT	0.774	0.695	0.765
ECFP6	SVM	0.971	0.935	0.982
	NB	0.864	0.885	0.889
	MLP	0.921	0.911	0.963
	LR	0.937	0.920	0.949
	RF	0.929	0.887	0.976
	DT	0.795	0.754	0.825
MACCS	SVM	0.935	0.891	0.972
	NB	0.577	0.585	0.598
	MLP	0.954	0.834	0.947
	LR	0.848	0.835	0.824
	RF	0.903	0.902	0.968
	DT	0.759	0.756	0.871
Molecular Descriptors	SVM	0.628	0.677	0.731
	NB	0.581	0.568	0.543
	MLP	0.899	0.837	0.937
	LR	0.811	0.824	0.789
	RF	0.907	0.894	0.959
	DT	0.743	0.740	0.821
Atom-pair & Molecular Descriptors	SVM	0.642	0.729	0.777
	NB	0.687	0.712	0.604
	MLP	0.927	0.879	0.944
	LR	0.937	0.910	0.946
	RF	0.911	0.903	0.969
	DT	0.744	0.716	0.843
ECFP6 & Molecular Descriptors	SVM	0.638	0.706	0.762
	NB	0.874	0.897	0.871
	MLP	0.970	0.898	0.959
	LR	0.938	0.911	0.965
	RF	0.917	0.902	0.978
	DT	0.732	0.734	0.821
MACCS& Molecular Descriptors	SVM	0.629	0.691	0.739
	NB	0.630	0.609	0.604
	MLP	0.902	0.879	0.955
	LR	0.868	0.874	0.871
	RF	0.910	0.903	0.968
	DT	0.762	0.762	0.840
Atom-pair & ECFP6 & MACCS & Molecular Descriptors	SVM	0.660	0.733	0.813
	NB	0.794	0.823	0.724
	MLP	0.939	0.912	0.970
	LR	0.954	0.925	0.969
	RF	0.917	0.905	0.980
	DT	0.762	0.738	0.833
